# Noncatalytic functions of ISOAMYLASE 1 and 2 affect the proportion of insoluble and soluble α-polyglucans in maize

**DOI:** 10.1093/plcell/koaf220

**Published:** 2025-09-22

**Authors:** Tracie A Hennen-Bierwagen, Martha G James, Carter J Newton, Emily M Juhl, Ugo Cenci, Steven Ball, Christophe Colleoni, Stacie L Shuler, William F Tracy, Alan T Culbertson, Alan M Myers

**Affiliations:** Roy J. Carver Department of Biochemistry, Biophysics, and Molecular Biology, Iowa State University, Ames, IA 50011, USA; Roy J. Carver Department of Biochemistry, Biophysics, and Molecular Biology, Iowa State University, Ames, IA 50011, USA; Roy J. Carver Department of Biochemistry, Biophysics, and Molecular Biology, Iowa State University, Ames, IA 50011, USA; Roy J. Carver Department of Biochemistry, Biophysics, and Molecular Biology, Iowa State University, Ames, IA 50011, USA; Unité de Glycobiologie Structurale et Fonctionnelle, Université des Sciences et Technologies de Lille, UMR8576 CNRS-USTL, Cité Scientifique, Villeneuve d'Ascq Cedex 59655, France; Unité de Glycobiologie Structurale et Fonctionnelle, Université des Sciences et Technologies de Lille, UMR8576 CNRS-USTL, Cité Scientifique, Villeneuve d'Ascq Cedex 59655, France; Unité de Glycobiologie Structurale et Fonctionnelle, Université des Sciences et Technologies de Lille, UMR8576 CNRS-USTL, Cité Scientifique, Villeneuve d'Ascq Cedex 59655, France; Department of Agronomy, College of Agricultural and Life Sciences, University of Wisconsin, Madison, WI 53076, USA; Department of Agronomy, College of Agricultural and Life Sciences, University of Wisconsin, Madison, WI 53076, USA; Roy J. Carver Department of Biochemistry, Biophysics, and Molecular Biology, Iowa State University, Ames, IA 50011, USA; Roy J. Carver Department of Biochemistry, Biophysics, and Molecular Biology, Iowa State University, Ames, IA 50011, USA

## Abstract

Starch arose in chloroplast-containing species from a combination of prokaryotic and eukaryotic genes involved in the metabolism of soluble branched α-polyglucan, i.e. glycogen. Nonmutant plants entirely lack such soluble polymers and instead contain amylopectin in insoluble starch granules. The transition between soluble and insoluble branched α-polyglucans during plant evolution is not well understood. This study generated maize (*Zea mays* L.) lines exhibiting a gradually varying distribution between soluble α-polyglucan and starch in the endosperm. These chemotypes were determined by complexes of conserved α-(1→6)-glucosidases of the isoamylase class (ISA). Four independent spontaneous missense substitutions in the ISA1 subunit of these complexes each cause a distinct soluble/insoluble α-polyglucan ratio, even though all 4 ISA1 variants lack detectable catalytic activity. These substitutions are located near each other in a domain distant from the active site. A separate region of ISA1 binds its noncatalytic paralog ISA2. Removal of ISA2 from the ISA1 mutant lines conditions further variability in the proportions of soluble α-polyglucan and starch. Thus, the extent of precursor α-polyglucan crystallization is determined by aspects of the ISA complexes beyond enzymatic activity. Various arrangements of multiple glucan-binding sites in different forms of the ISA1/ISA2 assemblies are proposed to determine how those complexes interact with precursor polymers. In turn, structural organization of the polymers is proposed to influence their crystallization, independent of α-1,6-glucosidase activity. Gradual change from soluble α-polyglucan metabolism to starch metabolism is proposed as a selective advantage leading to ISA2 conservation despite its lack of a functional catalytic site.

## Teaser

Complexes containing isoamylase 1 and isoamylase 2 affect the distribution between soluble and insoluble α-polyglucans in maize endosperm in part by functions other than enzymatic catalysis.

## Introduction

Glucose units are stored in branched α-polyglucan polymers in the great majority of species, either in the hydrosoluble form glycogen or in insoluble, semicrystalline starch granules. Glycogen and the starch polymer amylopectin resemble each other in chemical structure, both consisting of α(1→4)-linked linear chains of glucose units connected by α(1→6) glycoside bonds, i.e. branch linkages (Smith and Zeeman 2020; Tetlow and Bertoft 2020; Apriyanto et al. 2022). The fine structure of these polymers varies so that glycogen is soluble in aqueous environments whereas amylopectin assumes a semicrystalline form in insoluble starch granules. Glycogen predominates across the evolutionary spectrum, whereas starch occurs essentially in only 1 monophyletic subdivision, the Archaeplastida. Thus, glycogen was the primordial mode of hexose phosphate storage in α-polyglucans, and this was converted into a starch system in a common ancestor of the Archaeplastida. Presumably, this was not an instantaneous change, rather, intermediate species would have contained both soluble and insoluble α-polyglucan. At some point only starch was present, and this progenitor then gave rise to 3 sublineages, namely the Rhodophyceae (red algae), the Glaucophyta (a small group of freshwater algal species), and the Chloroplastida (chloroplast-containing species, i.e. green algae and land plants) (Adl et al. 2005). A distinctive feature of latter group is location of starch granules within chloroplasts.

Conversion of glycogen biosynthesis into a starch system involved incorporation of an α(1→6)-specific glycoside hydrolase from a structurally defined subgroup of the α-amylase superfamily (Kuriki and Imanaka 1999; MacGregor et al. 2001) designated in the CAZy classification system as subfamily GH13_11 (http://www.cazy.org/GH13.html) (Stam et al. 2006; Moller et al. 2016; Drula et al. 2022). GH13_11 enzymes are ubiquitous in eubacteria where they function in glycogen degradation and are referred to variously as GlgX (named after the corresponding gene locus) or isoamylase (ISA). In eukaryotes, GH13_11 enzymes, designated as ISA, are found essentially only in Archaeplastida species. After horizontal gene transfer from a eubacterial donor, eukaryotic ISA evolved to modify soluble α-polyglucans to promote crystallization rather than completely degrade such polymers (Ball et al. 1996; Myers et al. 2000; Boehlein et al. 2023). The precursor polymers are synthesized by starch synthases (SS) and starch branching enzymes (SBE), multiple forms of which are conserved in Chloroplastida (Deschamps et al. 2008; Hennen-Bierwagen and Myers 2013; Smith and Zeeman 2020). Photosynthetic hexose phosphate production and carbohydrate storage in starch granules were synergistic factors in establishing the Chloroplastida lineage.

This hypothesis for the role of ISA is founded on genetic analyses of diverse species showing that ISA mutation results in redistribution of α-polyglucan from insoluble starch to soluble polymers similar in structure to glycogen, referred to as phytoglycogen (James et al. 1995; Mouille et al. 1996; Zeeman et al. 1998; Kubo et al. 1999; Burton et al. 2002; Fujita et al. 2003; Hussain et al. 2003; Bustos et al. 2004; Lin et al. 2013). Analogous results were observed in 1 of the rare starch-accumulating cyanobacterial species (Cenci et al. 2013) and in a red alga (Maeno et al. 2022). The biosynthetic function of eukaryotic ISA is not essential for formation of starch because some level of insoluble α-polyglucan remains in mutants or in vivo reconstituted systems devoid of any such enzyme (Zeeman et al. 1998; Streb et al. 2008; Boehlein et al. 2023). Rather, ISA function appears to substantially increase the propensity of precursor branched polymers synthesized in the soluble phase to crystallize.

Three ISA paralogs arose through gene duplication in a common ancestor of the Chloroplastida, and have remained conserved (Deschamps et al. 2008). ISA3 retained the catabolic function and substrate specificity of the progenitor eubacterial GlgX (Wattebled et al. 2005; Delatte et al. 2006; Kobayashi et al. 2016). In contrast, ISA1 has a different substrate specificity and provides a biosynthetic function because mutation of this enzyme conditions the aforementioned decrease in starch content and appearance of phytoglycogen. The role of ISA2 is presently unclear because (i) ISA2 is not present in Rhodophyceae and Glaucophyta and thus is not essential for starch metabolism, (ii) ISA2 mutations cause varying effects in different species and tissues, and (iii) ISA2 appears to be noncatalytic owing to variation at residues directly involved in the hydrolysis mechanism (Hussain et al. 2003; Kubo et al. 2010; Sundberg et al. 2013). Why a noncatalytic version of ISA would continue to be selected is not understood. Potentially ISA2 could provide a function that influences α-polyglucan structure that is not dependent on inherent α(1→6)-glucosidase activity.

Regarding genetic effects, in some contexts ISA2 mutation conditions an essentially identical phenotype as that caused by ISA1 deficiency, indicating the 2 paralogs function together at the same point in α-polyglucan biosynthesis (Zeeman et al. 1998; Delatte et al. 2005; Wattebled et al. 2005). In other contexts, loss of ISA2 has no obvious effect on starch or phytoglycogen accumulation and thus appears to be dispensable (Kubo et al. 2010; Utsumi et al. 2011). In yet other situations ISA2 mutation conditions starch decrease and phytoglycogen accumulation but to lesser extents than the changes caused by ISA1 defects (Mouille et al. 1996; Dauvillee et al. 2001a, 2001b; Lin et al. 2013; Sim et al. 2014).

Part of the explanation for why some eukaryotic ISAs function in starch biosynthesis rather than α-polyglucan degradation may be their assembly into quaternary structures. Eubacterial GlgX generally functions as a monomer (Ray 2011). In contrast, multiple quaternary complexes are evident in cereals, including several forms of ISA1/ISA2 heteromultimer, and an ISA1/ISA1 homodimer (Utsumi and Nakamura 2006; Kubo et al. 2010; Facon et al. 2013; Lin et al. 2013; Trimble et al. 2016). *Chlamydomonas* also contains ISA1/ISA2 heteromultimers and an ISA1 homodimer (Mouille et al. 1996; Dauvillee et al. 2001a, 2001b; Sim et al. 2014). Arabidopsis leaves contain exclusively ISA1/ISA2 heteromeric complexes (Sundberg et al. 2013). The enzymatic consequences of the various multisubunit ISA assemblies are not yet known.

This study investigated ISA function by a mutational analysis in maize (*Zea mays* L.) using alleles that cause appearance of phytoglycogen. Soluble α-polyglucan does not occur in nonmutant plants. Numerous alleles of the maize gene encoding ISA1, *sugary1* (*su1*), have been characterized owing to an obvious kernel morphology phenotype that results from appearance of phytoglycogen and diminished starch content. Such alleles are present in maize land races owing to their centuries-long use in sweet corn lines (Whitt et al. 2002; Tracy et al. 2006). An a priori hypothesis predicts that the starch/phytoglycogen would correlate with catalytic activity of the ISA complexes. To the contrary, alleles that each cause apparently complete loss of enzymatic activity condition wide variation in the carbohydrate storage phenotype. Eliminating ISA2 in lines containing specific *su1-*alleles conditioned further changes in the starch/phytoglycogen ratio. The results indicate that the ISA function that stimulates conversion of soluble α-polyglucan precursors into semicrystalline amylopectin does not solely depend on catalytic properties. Continued evolutionary selection of ISA2, despite its lack of catalytic function, may have been based on modulation of the starch/phytoglycogen ratio during the transition of starch metabolism into plastids from its original location in the cytosol. Structural modeling suggests that the observed chemotypic variation in the absence of catalytic activity results from modular assembly of multiple α-polyglucan binding sites within ISA1/ISA2 complexes.

## Results

### Distinctive features of ISA2 structure

**Figure 1.**
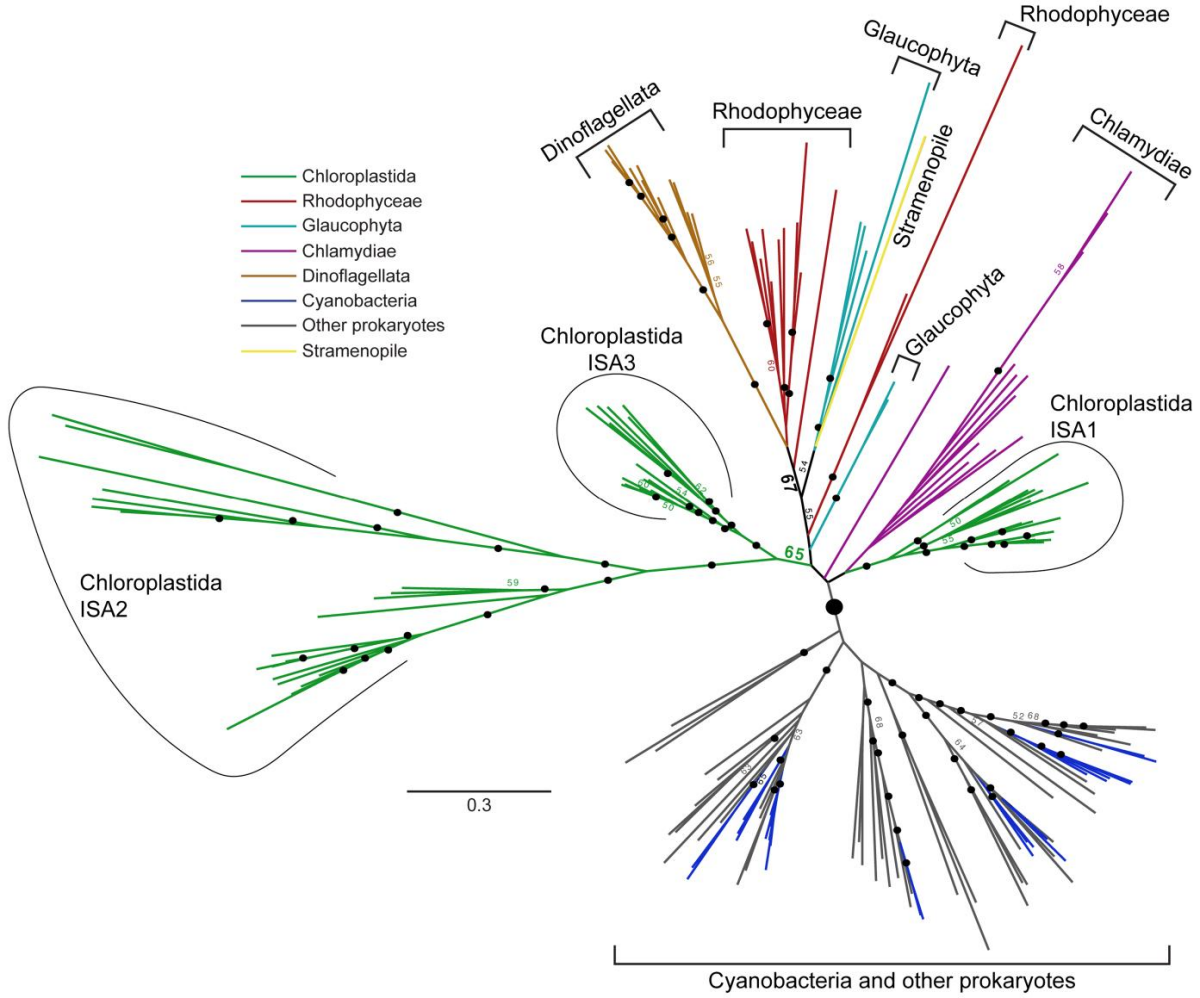
Unrooted ISA phylogenetic tree. Bootstrap values were determined from 100 repetitions. The large black dot indicates a bootstrap value of 95. Bootstrap values of >70 are indicated by small black dots, and values >50 are indicated numerically. Bootstrap values <50 are not shown. The scale bar shows the inferred number of amino acid substitutions per site. A rooted tree with species and all bootstrap values indicated is shown in Supplementary Fig. S1.

Phylogenetic analyses examined whether noncatalytic substitutions in the active site of ISA2, compared with other members of the α-amylase superfamily, are strictly conserved throughout the Chloroplastida lineage. Nontargeted phylogenetic analysis determined the relatedness of ISA2 throughout the Archaeplastida and to eubacterial GH13_11 proteins (Fig. 1) (Supplementary Fig. S1). Presence of 3 ISA paralogs is specific to the Chloroplastida, whereas Rhodophyceae and Glaucophyta contain only 1 ISA gene or in rare instances 2 paralogs apparently derived from relatively recent duplication (Maeno et al. 2022). A high-confidence bootstrap value separates all eubacterial GlgX or ISA from Archaeplastida ISA, except for proteins from numerous Chlamydiae species that group in the same clade as the photosynthetic eukaryotes (Fig. 1) (Supplementary Fig. S1). This expanded analysis provides strong support for the conclusion that ISA2 existed in a common ancestor of the Chloroplastida and has been retained throughout subsequent evolution. ISA2, however, has a comparatively elevated rate of sequence change compared with other monophyletic ISA homologs, as indicated by branch lengths (Fig. 1) (Supplementary Fig. S1). The analysis also strongly supports the previous conclusion that the eubacterial source of eukaryotic ISA was a Chlamydiae species (Becker et al. 2008; Moustafa et al. 2008; Ball et al. 2013; Maeno et al. 2022).

**Figure 2.**
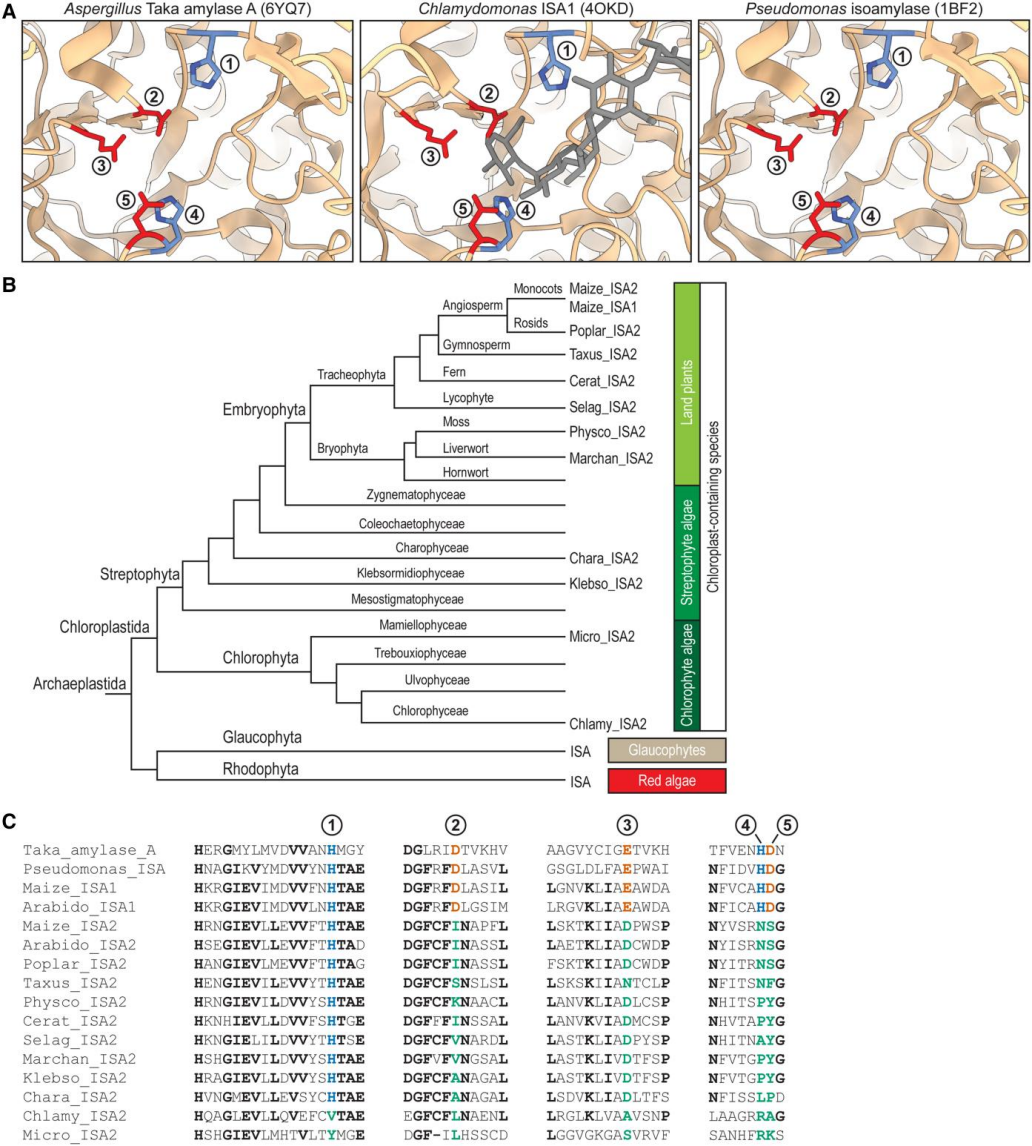
ISA2 sequence divergence at catalytic residue sites. **A)** Structures of the catalytic site of α-amylase superfamily (GH13) enzymes. Protein Data Bank coordinate files are indicated in parentheses. Residues in red or blue color are essentially universally conserved in GH13 proteins and are directly involved in catalysis or substrate binding, respectively. Gray color indicates substrate analogs determined in cocrystals. Circled numerals correspond to panel **(C)**. **B)** Conceptualized Archaeplastida phylogenetic tree indicating the position of species analyzed in panel **(C)** (from Leebens-Mack et al. 2019). **C)** Sequences of ISA2 proteins and representative α-amylase superfamily enzymes in the regions containing the conserved catalytic residues. Circled numerals correspond to panel **(A)**. Blue text indicates correspondence to residues colored blue in panel **(A)**, and vermillion text indicates correspondence to residues colored red in panel **(A)**. Bluish green text indicates residues that vary from the consensus active site residues. Bold black text indicates residues conserved to a high degree in ISA2 proteins. Genbank identifiers and species are: *Aspergillus oryzae* Taka_amylase_A, BAA00336; *Pseudomonas amyloderamosa* Pseudomonas_ISA, P10432; *Zea mays* Maize_ISA1, XP_008678357; *Arabidopsis thaliana* Arabido_ISA1, AEC09752; *Zea mays* Maize_ISA2, PWZ18327; *Arabidopsis thaliana* Arabido_ISA2, NP_171830; *Populus alba* Poplar_ISA2, XP_034908173; *Taxus chinensis* Taxus_ISA2, KAH9320558; *Physcomitrella patens* Physco_ISA2, XP_024382383; *Ceratopteris richardii* Cerat_ISA2, KAH7435504; *Selaginella moellendorffi*i Selag_ISA2, XP_024535142; *Marchantia polymorpha* Marchan_ISA2, PTQ31894; *Klebsormidium nitens* Klebso_ISA2, GAQ80182; *Chara braunii* Chara_ISA2, GBG76493; *Chlamydomonas reinhardtii* Chlamy_ISA2, PNW69936; *Micromonas pusilla* Micro_ISA2, EEH59305.

Amino acid sequences were then analyzed to determine the evolutionary distribution of amino acid substitutions in ISA2 that apparently render it noncatalytic. ISA2 from several angiosperms was observed previously to vary at the positions of several well-characterized catalytic residues otherwise conserved throughout the GH13 superfamily (Hussain et al. 2003; Kubo et al. 2010; Sundberg et al. 2013) (Fig. 2A). This analysis was extended broadly throughout the Chloroplastida (Leebens-Mack et al. 2019), including streptophyte and chlorophyte algae as well as diverse land plants (Fig. 2B). At least 4 of the 5 conserved residues vary in every ISA2 protein examined (Fig. 2C), including the nucleophile that initiates glycoside bond hydrolysis (Fig. 2A, circled numeral 2). These observations indicate that a noncatalytic ISA paralog existed in the common ancestor of the Chloroplastida, and this protein was subject to evolutionary selection thereafter.

**Figure 3.**
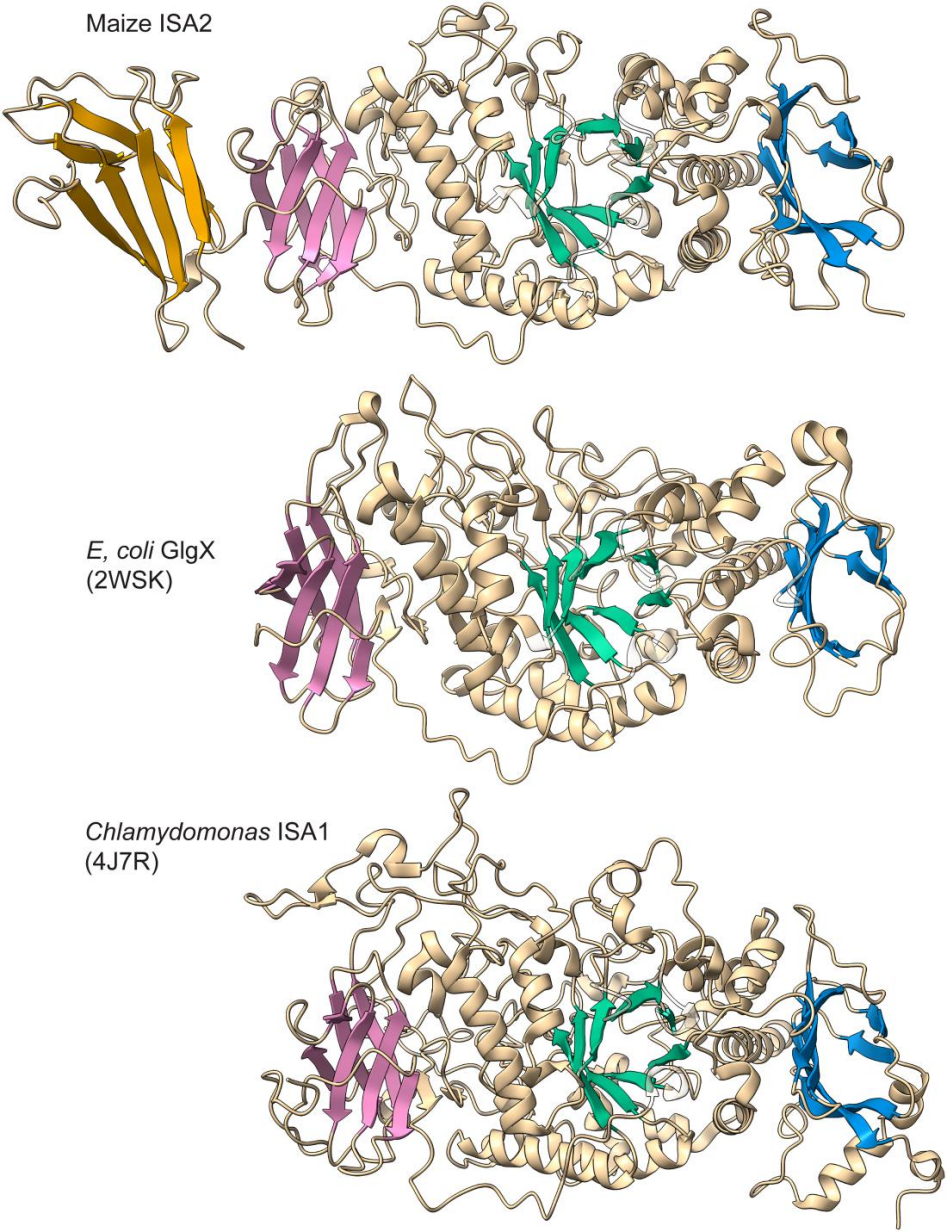
Maize ISA2 structure compared with known GH13_11 protein structures. The maize ISA2 primary sequence (Genbank accession number PWZ18327) was modeled using AlphaFold3. Protein Data Bank identifiers are indicated in parentheses. Bluish green color indicates the catalytic domain, reddish purple color indicates CBM48, blue color indicates a ß-sandwich domain present in all GH13 proteins, and orange color indicates a ß-sandwich domain specific to ISA2. Structures of additional ISA2 proteins and α-amylase superfamily members are shown in Supplementary Fig. S2.

Structural modeling with AlphaFold3 revealed another unique structural feature of ISA2 compared with other GH13_11 proteins. As expected from sequence homology, ISA2 is predicted to adopt the common tertiary structure observed directly for GH13_11 enzymes from archaeal or eubacterial species, as well as *Chlamydomonas* ISA1 (Fig. 3). GH13_11 proteins contain (i) a structural motif designated as carbohydrate binding module 48 (CBM48) (Janecek et al. 2011; Meekins et al. 2014), (ii) a (β/α)_8_ barrel containing the catalytic site, and (iii) a conserved C terminal β-sandwich domain. ISA2 is distinguished by an additional β-sandwich domain at the amino terminus that is not present in other ISAs or any other member of the α-amylase superfamily examined for this report (Fig. 3) (Supplementary Fig. S2). This domain is predicted in ISA2 from diverse Chloroplastida species, indicating it was present at the root of the lineage and has been retained subsequently. The N-terminal β-sandwich domain of ISA2 does not align closely with the adjacent CBM48 domain nor has it been identified as any other defined CBM domain in the CAZy database.

### Phenotypic variation conditioned by *su1-*alleles

**Figure 4.**
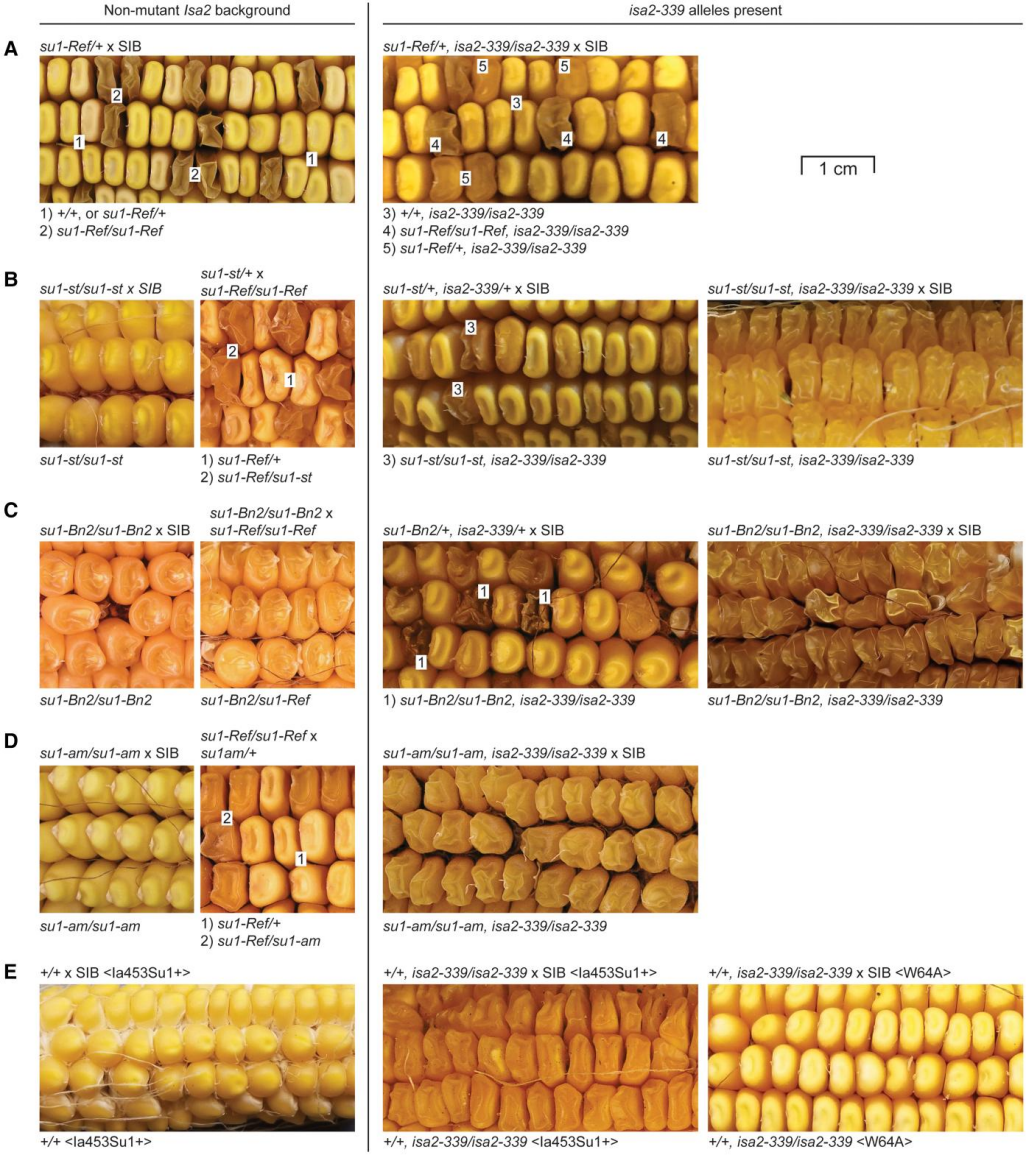
Kernel phenotypes. All ears are in the W64A background except for those in panel E indicated as in the Ia453Su1 + background. Genotypes of the parent plants and the progeny kernels are shown above and below the ear photograph, respectively. “+” indicates nonmutant alleles. “SIB” indicates a sibling plant of the same genotype as the other parent of the cross. The scale bar applies to all panels. **A)** Effects of *su1-Ref*. **B)** Effects of *su1-st*. **C)** Effects of *su1-Bn2*. **D)** Effects of su1-am. **E)** Effect of *isa2-339* in the Ia453Su1 + or W64A inbred backgrounds homozygous for a nonmutant *Su1* allele.

Four independent, spontaneous alleles of the *su1* locus were introgressed in the common inbred genetic background of dent corn line W64A (Supplementary Fig. S3). These are the missense mutations *su1-Ref* and *su1-am*, affecting residues 578 and 308, respectively, the transposon insertion allele *su1-st* that affects pre-mRNA splicing, and the mutant allele *su1-Bn2* that has not previously been characterized at the molecular level (Table 1). Each allele causes a distinct visual phenotype ranging from strongly shriveled and translucent kernels conditioned by *su1-Ref* (Fig. 4A) to apparently normal dent corn kernels resulting from *su1-am* (Fig. 4D). The intermediate kernel phenotypes conditioned by *su1-st* (Fig. 4B) or *su1-Bn2* (Fig. 4C) are distinct from each other and from the *su1-Ref* phenotype. The *su1-st* and *su1-Bn2* alleles differ also in that the former is recessive to *su1-Ref* regarding visual kernel phenotype (Fig. 4B) whereas the latter is dominant (Fig. 4C). The allele *su1-am*, which by itself causes no abnormal kernel phenotype, is also fully recessive to *su1-Ref* (Cameron 1947; Mangelsdorf 1947). These results reproduce data described previously in a conference report (Garwood and Creech 1972).

**Table 1.** Alleles of the su1 locus mentioned in this study

Allele	Mutation^a^	Effect^a^	Reference
*su1-Ref* (*su1-NE*)^b^	T to C transition at nt 6830	Replaces Trp 578 by Arg (W578R)	Correns 1901; Dinges et al. 2001; Tracy et al. 2006; Whitt et al. 2002
*su1-st*	Transposon after nt 3510	Altered pre-mRNA splicing, low protein level	Dahlstrom and Lonnquist 1964; Dinges et al. 2001
*su1-am*	G to T transition at nt 2541	Replaces Arg 308 by Ile (R308I)	Mangelsdorf 1947; Kubo et al. 2010
*su1-Bn2*	C to G transversion at nt 7626	Replaces Asn 628 by Lys (N628K)	Garwood and Creech 1972; This study
*su1-NC*	C to T transition at nt 6171	Replaces Arg 504 by Cys (R504C)	Tracy et al. 2006
*su1-SW*	A to G transition at nt 6780	Replaces Asn 561 with Ser (N561S)	Tracy et al. 2006
*su1-4582*	Transposon after nt 505	Null, no protein present	James et al. 1995

^a^Nucleotide and amino acid numbers refer to *Su1* transcript Zm00001eb174590-T001 annotated in the Zm-B73-REFERENCE-NAM-5.0 genome sequence (https://www.maizegdb.org).

^b^ *su1-NE* and *su1-Ref* are the same allele isolated separately from divergent populations.

**Figure 5.**
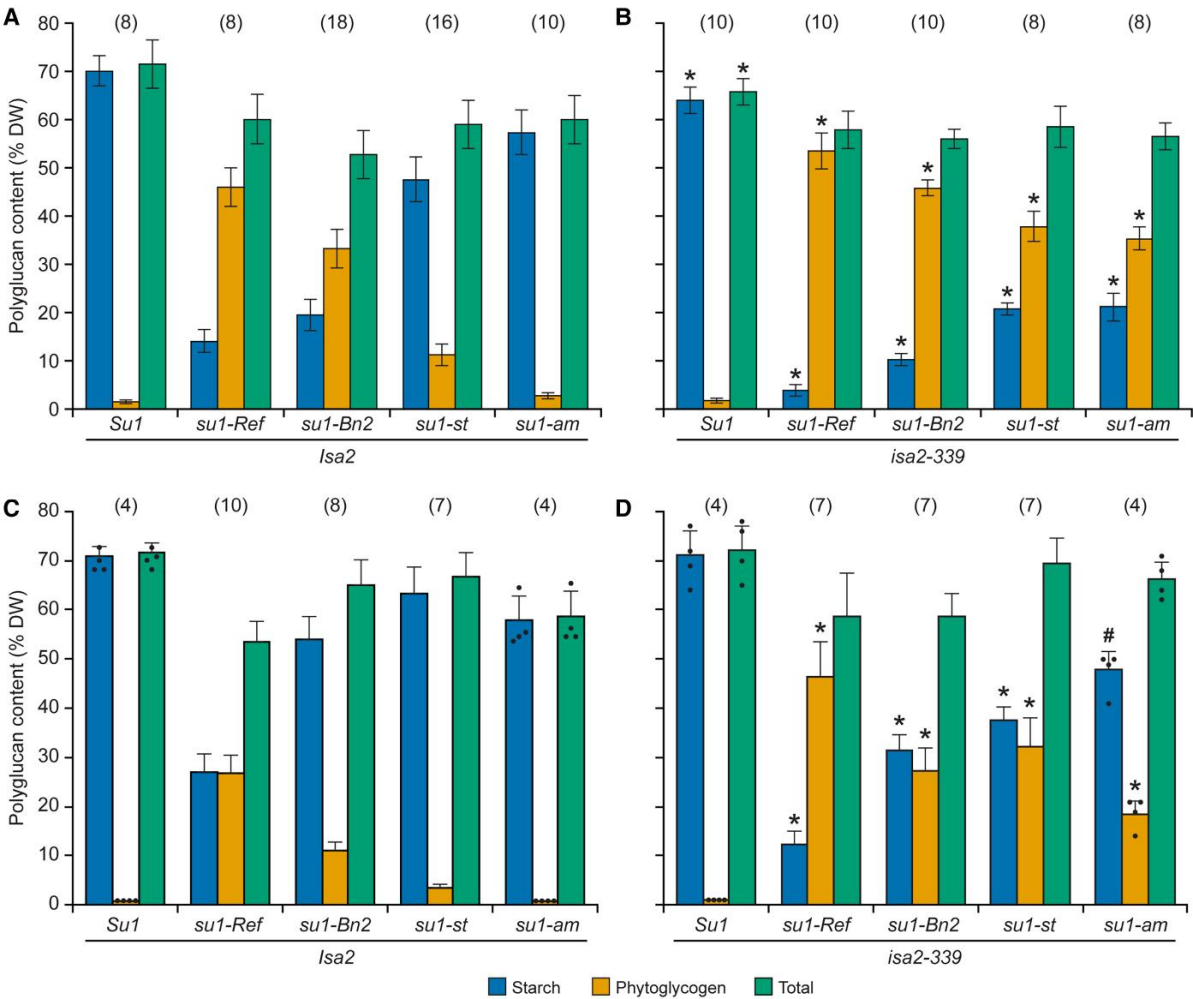
Endosperm starch and phytoglycogen content. Lines are homozygous for the indicated alleles in the W64A inbred background. *Su1* is the nonmutant allele from inbred W64A. Values are averages ± SD as a percentage of dry weight. The number of biological replicates used to determine each set of values is indicated in parentheses. Black dots indicate individual measurements. Statistical significance was determined by two-tailed Student's *t*-test (Supplementary Data Set 3). **A)** Immature endosperm harvested 20 DAP from lines homozygous for the nonmutant allele *Isa2*. **B)** Immature endosperm harvested 20 DAP from lines homozygous for the null allele *isa2-339*. Asterisks (*) indicate significant difference from the corresponding *Isa2/Isa2* strain shown in panel **(A)** (*P* value < 0.01). **C)** Mature endosperm from lines homozygous for *Isa2*. **D)** Mature endosperm from lines homozygous for *isa2-339*. Asterisks (*) indicate significant difference from the corresponding *Isa2/Isa2* strain shown in panel **(C)** at *P* value < 10^−4^ and the pound sign (#) indicates significant difference from the corresponding *Isa2/Isa2* strain at *P* value < 0.03.

Starch and phytoglycogen content as a percentage of dry weight was determined in endosperm tissue at mid-development, 20 days after pollination (DAP) (Fig. 5A). As expected, α-polyglucan in the nonmutant standard is entirely in the starch granule fraction. Background signal from the soluble fraction of the nonmutant control is derived from low molecular mass maltooligosaccharides rather than molecules with a high degree of polymerization. Total α-polyglucan content conditioned by any of the 4 *su1*-alleles was significantly reduced 16% to 25% from the nonmutant level (*P* value < 0.001). Total polymer, however, did not vary substantially between any of the 4 mutant lines. The distribution between phytoglycogen and starch was unique in each of the 4 mutants (Fig. 5A). Soluble and insoluble α-polyglucan content were each significantly different in every instance in pairwise comparisons between genotypes (*P* value < 0.001).

Similar results were observed in endosperm from mature kernels (Fig. 5C). Total α-polyglucan was again moderately reduced in the mutants compared with the wild type control. Each allele again resulted in a distinct distribution between starch and phytoglycogen. All values for starch and phytoglycogen content varied significantly in pairwise comparisons between *su1-Ref*, *su1-Bn2*, and *su1-st* endosperm (*P* value < 0.007), and *su1-am* tissue varied from the other mutants by lacking phytoglycogen entirely. Phytoglycogen content correlated with severity of the visual kernel phenotype (Fig. 4). During development from 20 DAP to maturity the normalized phytoglycogen content decreased, and the starch level increased (Fig. 5, A and C).

### Molecular characterization of *su1-Bn2*

Changes in the ISA1 primary sequence caused by *su1-Ref*, *su1-st*, or *su1-am* are known (Table 1), but the molecular nature of *su1-Bn2* has not been described. After introgressing *su1-Bn2* into the W64A background, the 8.37 kb sequence of the near-complete transcribed region of the gene was determined from overlapping PCR-amplified fragments. The *su1-Bn2* allele arose in the *su1* haplotype shared by the great majority of dent corn lines (designated as the “field corn haplotype”), in contrast to *su1-Ref* that arose in a distinct haplotype shared by almost all sweet corn, teosinte, flint corn, and popcorn lines (designated as the “sweet corn haplotype”) (Hu et al. 2021) (Supplementary Fig. S4). Within the field corn haplotype, a subgroup that includes inbred Oh43 matches the inbred B73 reference sequence (https://www.maizegdb.org, gene model Zm00001eb174590) except for a single nucleotide polymorphism (SNP) that changes codon 662 from Lys in B73 to Glu in Oh43 (K662E). The *su1-Bn2* allele matches the genomic sequence of Oh43 exactly, except for 1 additional SNP that changes codon 628 from Asn in Oh43, and every other characterized maize inbred, to Lys. Homozygosity at this SNP cosegregated with the distinctive kernel phenotype imparted by *su1-Bn2* in crosses between *su1-Bn2/+* heterozygotes. These data identify the Asn to Lys substitution at residue 628 (N628K) as the causative agent of *su1-Bn2*.

### Effects of *su1-*alleles on ISA1 expression

**Figure 6.**
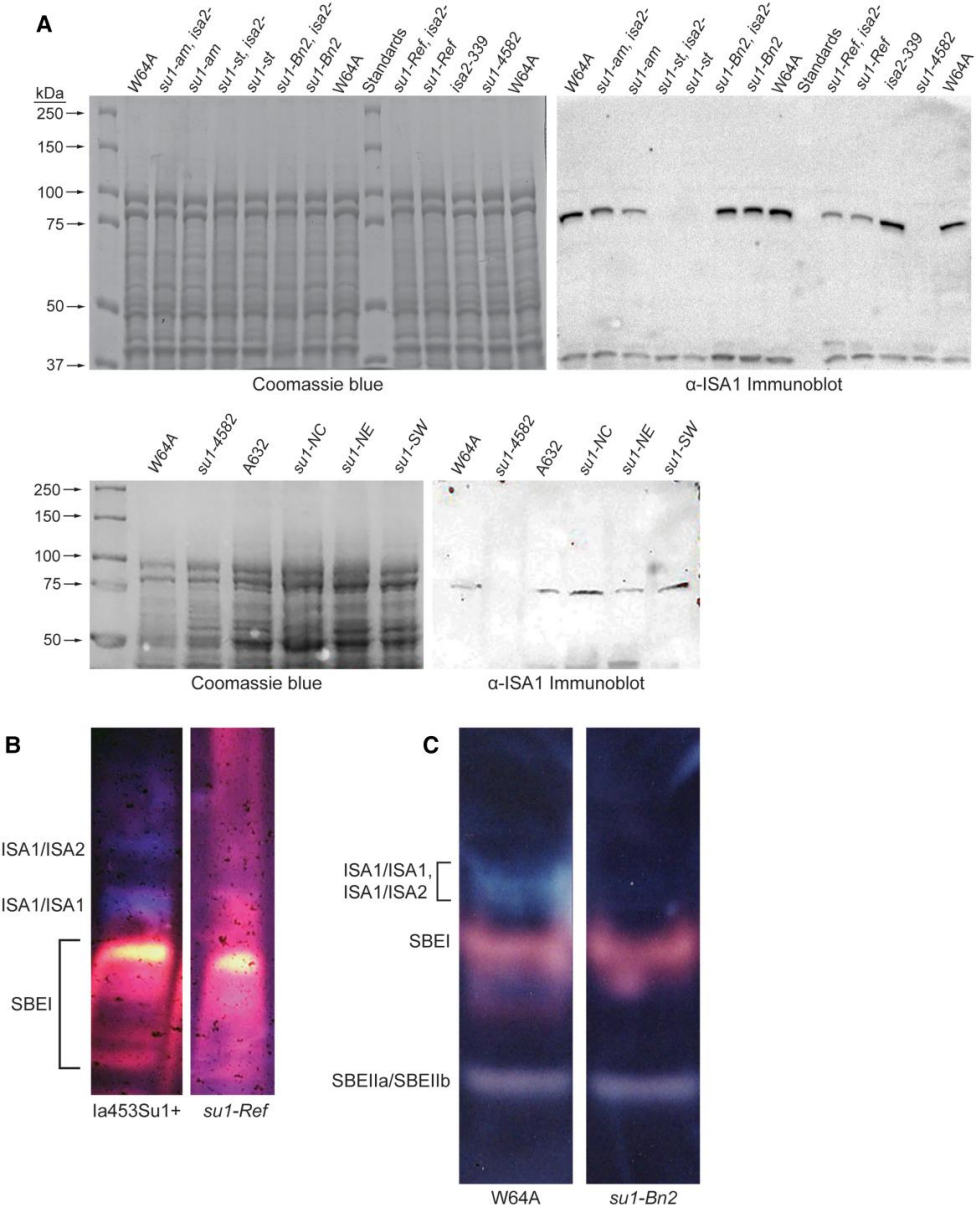
ISA1 protein and enzyme expression in endosperm extracts. Allele names indicate endosperm homozygous for that mutation.”*isa2-*” indicates the null allele *isa2-339*. **A)** Immunoblot analysis. Total soluble endosperm extracts (40 μg) from kernels of the indicated homozygous genotype, harvested mid-development (20 DAP), were fractionated by SDS-PAGE, then duplicate gels were stained with Coomassie blue or probed with anti-ISA1 IgG. W64A and A632 are nonmutant standards. **B)** In-gel enzyme activity assay, 15 cm gels, 50 μg total soluble extract. Ia453Su1 + is the nonmutant standard (*Su1/Su1*) and “*su1-Ref*” is a homozygous mutant congenic in that background. **C)** In-gel enzyme activity assay, 6 cm gels, 25 μg total soluble extract. “W64A” is the nonmutant standard (*Su1/Su1*) and “*su1-Bn2*” is a homozygous mutant congenic in that background. The constituents of each activity band in panels **(B)** and **(C)** were identified previously (Colleoni et al. 2003; Kubo et al. 2010). In the 6 cm gels in Panel **(C)** all 3 forms of ISA1 activity seen in Panel **(B)** compress into a single band.

Immunoblot analysis of 20 DAP endosperm extracts compared the in vivo steady state levels of the normal and mutant ISA1 proteins (Fig. 6A). The antibody probe is an affinity purified IgG fraction (α-ISA1), raised against an ISA1-specific synthetic peptide. Specificity of α-ISA1 was demonstrated by detection of a protein matching the known electrophoretic mobility of native ISA1 in nonmutant extracts, and absence of that signal from tissue homozygous for the null allele *su1-4582*. As seen previously (Kubo et al. 2010) the mutant protein encoded by *su1-Ref*, ISA1*-*W578R, accumulates to a reduced but appreciable level compared with wild type ISA1. The *su1-am* product, ISA1-R308I, is also clearly detectable although less so than wild type. The *su1-Bn2* product, ISA1-N628K, accumulates at approximately wild type levels. ISA1 was not detected in tissue homozygous for *su1-st*, which is caused by *Mu* transposon insertion in exon 10.

Additional spontaneous *su1-*mutations extant in sweet corn breeding populations were included in this analysis (Table 1). The *su1-NC* and *su1-SW* mutations code for the missense variants ISA1-R504C or ISA1-N562S, respectively (Tracy et al. 2006). Both mutations condition decreased endosperm starch content and accumulation of phytoglycogen, and produce mutant forms of ISA1 that accumulate to approximately wild type levels (Trimble et al. 2016). Observation of nonmutant levels of these 2 ISA1 variants was reproduced here by immunoblot analysis (Fig. 6A). A third spontaneous allele isolated from sweet corn populations, *su1-NE*, is an independent isolate of the same mutation designated as su1-Ref (Tracy et al. 2006). Immunoblot analysis again revealed accumulation of the corresponding missense variant, ISA1-W578R, at reduced levels compared with wild type (Fig. 6A).

### ISA1 missense variants lack catalytic activity

ISA activity can be visualized after native-PAGE by in-gel enzyme assay in which light blue bands on dark backgrounds of iodine-stained starch. Three such bands typically are present in analysis of nonmutant endosperm, and all of these are absent from *su1-4582* null mutants (Dinges et al. 2001; Kubo et al. 2010). All 3 activity bands are missing from *su1-Ref* mutants (Dinges et al. 2001; Kubo et al. 2010), and that result was reproduced here in a different inbred genetic background as a control (Fig. 6B). ISA1 activity appeared to be completely missing from *su1-Bn2* endosperm (Fig. 6C), even though the mutant protein, ISA1*-*N628K, accumulates to normal levels (Fig. 6A). The ISA1 variants produced from *su1-NC* and *su1-SW*, which also accumulate in endosperm to approximately wild type levels (Fig. 6A), previously were also shown to be devoid of ISA activity in the in-gel enzyme assay (Trimble et al. 2016). Thus, the general characteristic of accumulation in endosperm at mid-development of an apparently catalytically inactive form of ISA1 has been observed repeatedly for 4 independent missense mutations.

**Figure 7.**
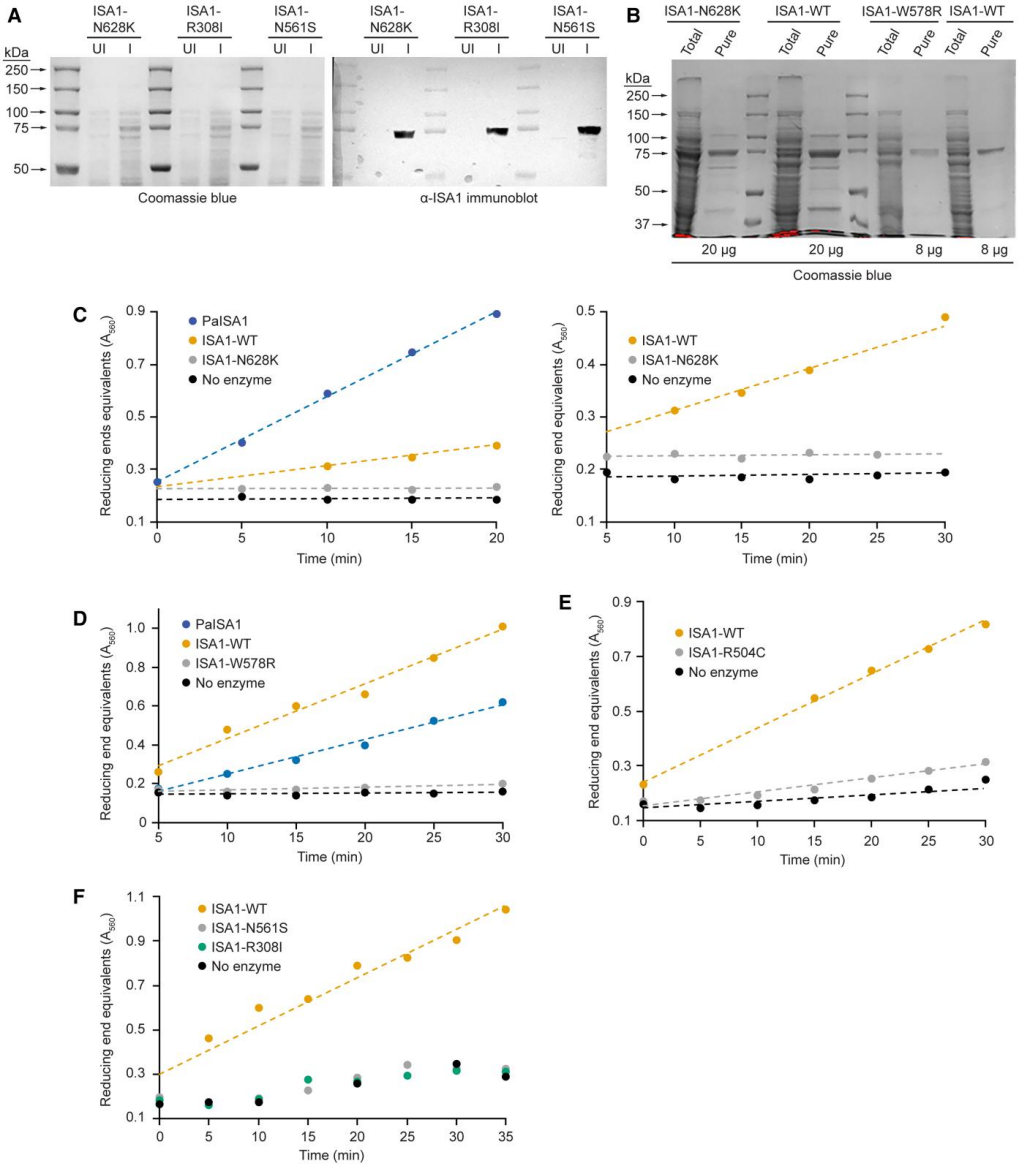
Recombinant expression and activity of ISA1 variants. **A)** Expression of ISA1 variants in total *E. coli* extracts. Total cell lysates from uninduced (UI) and induced (I) cell cultures were separated by SDS-PAGE and probed with α-ISA1 IgG. **B)** Purification. The Ni-agarose eluate peak containing ISA1 was concentrated, and the indicated amount of total protein was analyzed by SDS-PAGE and Coomassie blue staining. “Total” is the soluble *E. coli* lysate applied to the column, and “Pure” is the peak fraction eluted at 120 mm imidazole. **C**-**F**) α-(1→6)-glucosidase activity. Enzyme sources are 10 to 20 μg of concentrated Ni-agarose fractions shown in Panel **(B)**, with volumes adjusted for constant enzyme concentration in each assay. Data points are individual measurements and dashed lines are least squares best fit to those points. Each plot is data from a unique ISA1 preparation starting from a separate *E. coli* transformation. Enzymes were assayed on the same day they were purified from *E. coli*, except for the nonmutant ISA1-WT in panel **(C)**, which was stored overnight at 0 °C. The 2 plots in Panel **(C)** are from the same assay. PaISA1 is commercial Pseudomonas ISA. Table 1 shows the correspondence between ISA1 variant and *su1-*allele.

Potential enzymatic activity of wild type and mutant ISA1 variants was tested further using partially purified recombinant enzymes expressed in *E. coli*. Six ISA1 sequences were expressed that vary from each other only at a single amino acid within 740 residues. These are the nonmutant form, ISA1-WT, and each of the mutant forms encoded by *su1-Ref*, *su1-am*, *su1-Bn2*, *su1-NC*, or *su1-SW* (Table 1). The amino terminus of the recombinant proteins matched the mature in vivo N terminus (Rahman 1998) and the C terminus was fused to 8 successive His residues. ISA proteins were tracked during Ni-affinity purification by immunoblots using α-ISA1 IgG (Fig. 7A). ISA1 was the predominant protein in the affinity purification fractions (Fig. 7B).

These fractions were subjected to enzyme assays that quantified α(1→6)*-*glucosidase activity as reducing ends formed over time, normalized to protein content, with oyster glycogen as the substrate (Fig. 7, C to F). The assay sets performed each day included commercial *Pseudomonas* ISA as a positive control and a maltose standard curve to quantify reducing end concentration. The nonmutant ISA1 enzyme preparation stored overnight at 0 °C (Fig. 7C) exhibited less activity than “fresh” ISA1-WT preparations, i.e. those assayed on the same day as purification (Fig. 7, D to F). Thus, all subsequent assays were performed on freshly purified proteins.

The nonmutant protein ISA1-WT reproducibly exhibited substantial α(1→6) glucosidase activity in multiple independent preparations of the enzyme (Fig. 7, C to F). None of the mutants generated new reducing ends at a level above the negative control lacking enzyme (Fig. 7, C to F). Thus, all 5 of the recombinant mutant ISA1 proteins lack detectable enzymatic activity. Whether some trace level of activity remains that is below the level of detection cannot be ruled out, and in fact there may be some trace activity above background for ISA1-R504C (Fig. 7E). Nonetheless, in all cases the activity of the mutant protein is at or close to the zero value when background is subtracted, and far lower than that of the wild type variant.

### Elimination of ISA2 in *su1-*mutant backgrounds—kernel and carbohydrate phenotypes

Double mutant lines were generated that are homozygous for specific *su1-*alleles and lack ISA2 owing to homozygosity of the *isa2-339* transposon insertion allele (Supplementary Fig. S3). Loss of ISA2 in *su1-st*, *su1-Bn2*, or *su1-am* homozygous mutant backgrounds caused noticeable change in kernel phenotype, indicative of increased phytoglycogen content (Fig. 4, B to D). Eliminating ISA2 in the *su1-Ref* homozygous background did not cause a noticeable change in the kernel phenotype because *su1-Ref* by itself conditions a severely shrunken and translucent appearance (Fig. 4A).

The effects of ISA2 deletion on starch and phytoglycogen content were compared in congenic lines that vary only by the *su1* allele. Eliminating ISA2 in the otherwise nonmutant W64A background did not alter the kernel phenotype (Fig. 4E), nor did it condition accumulation of phytoglycogen or affect total α-polyglucan content either at 20 DAP or maturity, confirming previous results (Fig. 5, B and D) (Kubo et al. 2010). In 4 separate *su1-*mutant backgrounds, loss of ISA2 had significant effects. Total α-polyglucan content did not vary significantly between any pair of congenic single- and double-mutants. In each instance, loss of ISA2 resulted in significantly less starch and more phytoglycogen compared with the *su1-*single mutant. This effect was evident both at mid-development (Fig. 5, A and B) and maturity (Fig. 5, C and D). These data demonstrate that nonmutant ISA2 protein enhances starch accumulation in conditions when ISA1 catalytic function is absent.

**Figure 8.**
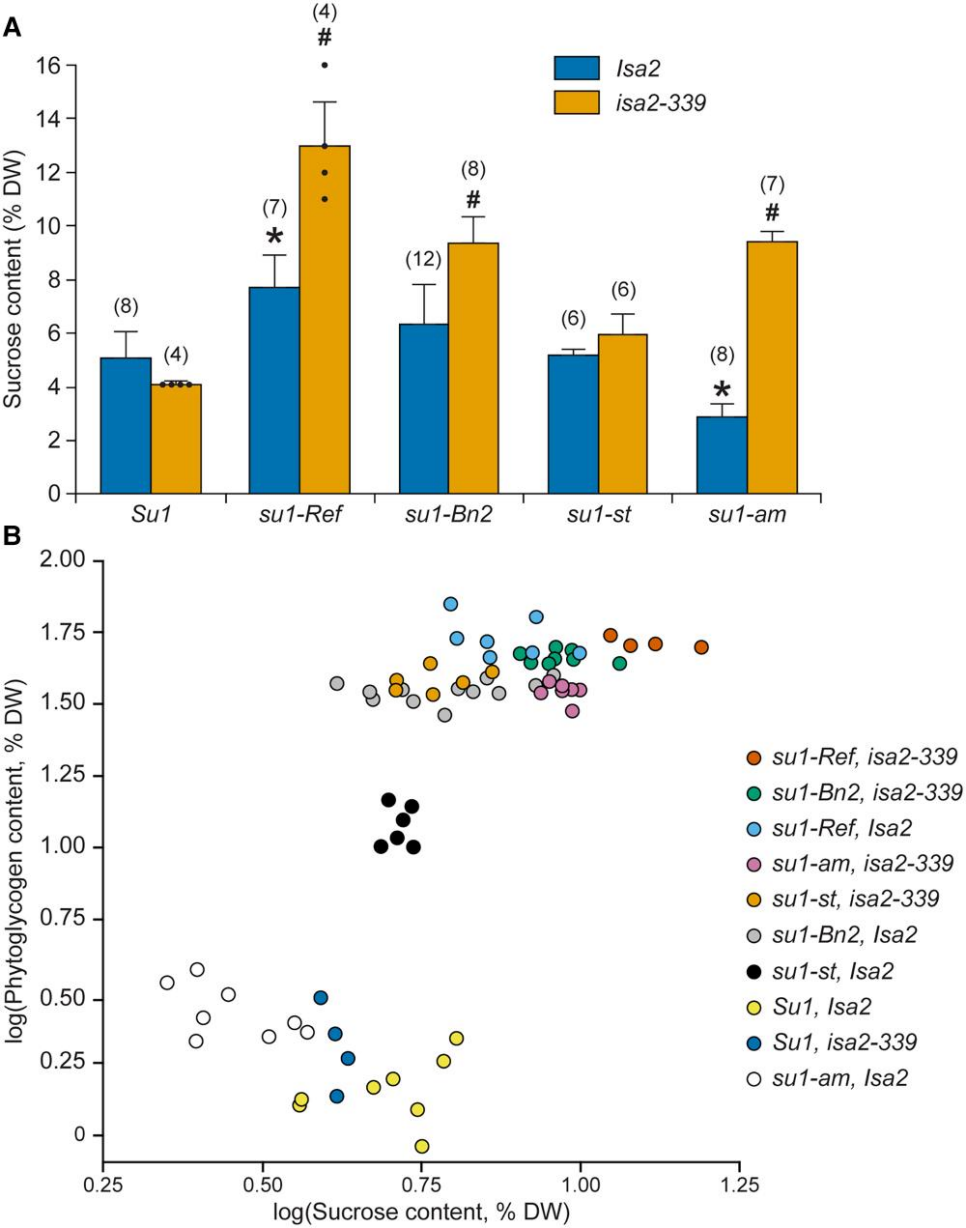
Sucrose content. **A)** Sucrose content in endosperm harvested 20 DAP. Values are average ± SD as a percentage of dry weight. Endosperms are homozygous for the indicated alleles at the *su1* and *isa2* loci. “*Su1*” and “*Isa2*” are the nonmutant alleles from inbred W64A. The number of biological replicates used to determine each value is indicated in parentheses. Black dots indicate individual measurements. Asterisks (*) indicate significant difference from the congenic *Su1* standard and pound signs (#) indicate significant difference from the congenic *Isa2* strain (two-tailed Student's *t*-test, *P* value < 0.001) (Supplementary Data Set 4). **B)** Correlation between phytoglycogen and sucrose content. Each point indicates data from a single endosperm.

The *isa2-*null mutation was also examined for effects on sucrose content in immature kernels in the series of congenic lines (Fig. 8A). The sucrose level in nonmutant W64A and the *isa2-339* single mutant did not vary significantly. As expected, sucrose was significantly elevated in *su1-Ref* single mutants. Other *su1-*alleles, however, did not condition elevated sucrose content in single mutant lines. Loss of ISA2 resulted in significantly elevated sucrose content compared with near-isogenic *su1-*single mutants in lines homozygous for *su1-Ref*, *su1-Bn2*, or *su1-am*. Variation in both sucrose and phytoglycogen content in these congenic lines enabled testing for correlation between these 2 carbohydrates. Sucrose levels were moderately correlated with phytoglycogen content across all genotypes (Spearman's ρ = 0.73, *P* < 0.001) (Fig. 8B).

### Elimination of ISA2 in *su1-*mutant backgrounds—effects on α-polyglucan structure

**Figure 9.**
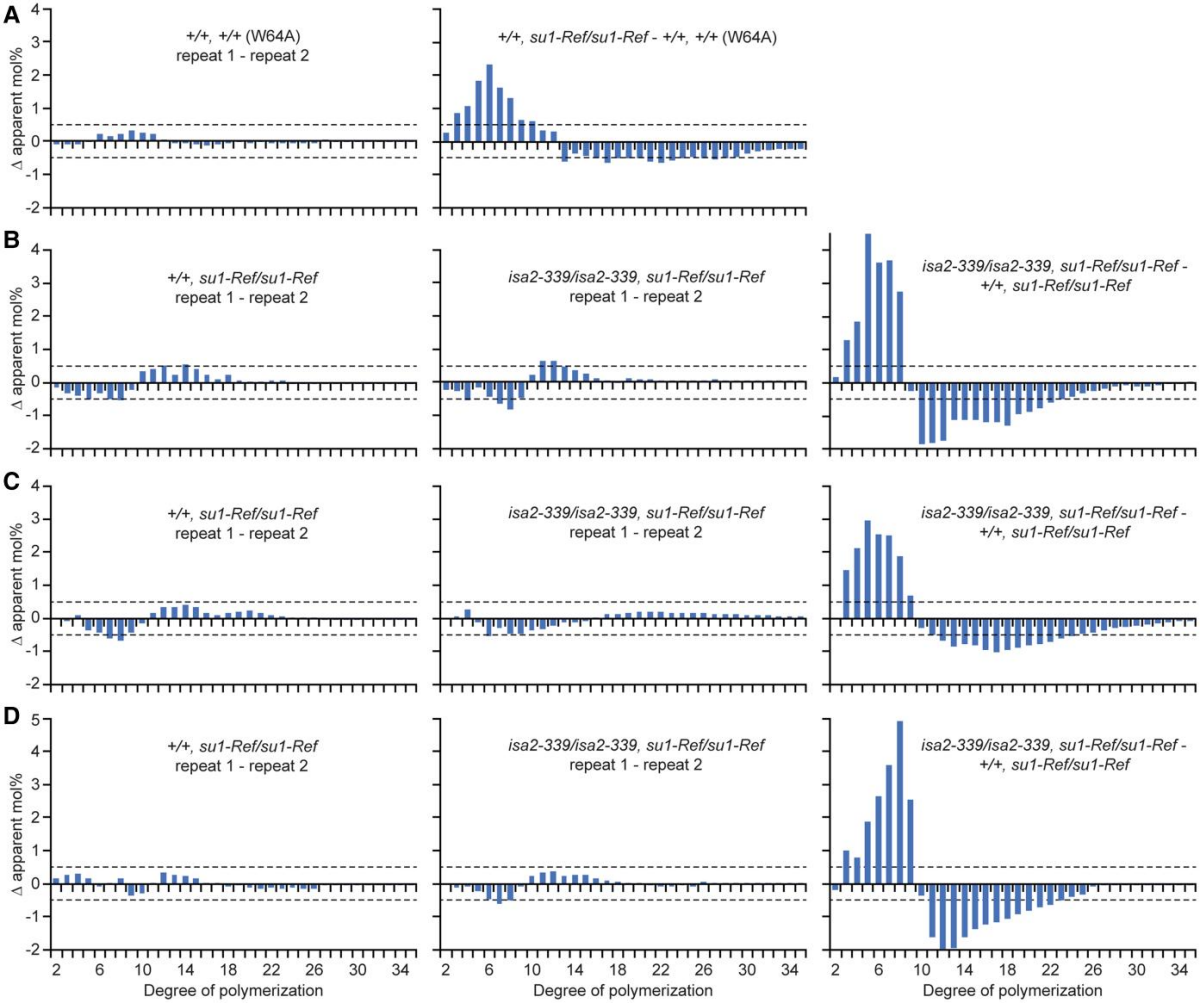
Amylopectin linear chain length distribution differences in *su1-ref* endosperm. All kernels are in the W64A inbred background. Repeats are biological replicate analyses of separate isogenic single kernels. The average of the 2 biological replicates was used to compare between genotypes. Dotted lines indicate 0.5% difference, assigned as the limit of technical variation. All plots are at the same scale. **A, B)** Mature kernels grown on separate ears, 2021 field season. **C)** Mature sibling kernels grown on the same ear, 2018 field season. **D)** Immature kernels harvested 20 DAP grown on separate ears, 2020 field season. Additional biological repeats of this experiment are shown in Supplementary Fig. S5.

The *su1-Ref* mutation results in altered structure of amylopectin within remnant starch granules, specifically the frequency distribution of α(1→4)-linked linear chain lengths. In the mutant amylopectin the abundance of linear chains containing 5 to 11 glucosyl units, i.e. degree of polymerization (DP) 5 to 11, is increased compared with wild type amylopectin, with compensating decreased frequency of longer chains (Dinges et al. 2001). That result was reproduced here as a control (Fig. 9A). Congenic lines were then used to examine whether loss of ISA2 further affects amylopectin linear chain length distribution in the *su1-Ref* background. Reproducibility of the assay was demonstrated by comparing biological replicate samples of the same genotype. Variation for each chain length between biological replicates was <0.5% for most chain lengths, which is taken as background technical variation (Fig. 9).

Amylopectin from mature double mutant endosperm lacking ISA2 was markedly increased in abundance of chains ranging from DP 3 to 9 compared with the *su1-Ref* single mutant, and correspondingly decreased in longer chain abundance ranging to approximately DP 25. This chemotype cosegregates with ISA2 deletion. The same effect was observed repeatedly over 3 generations, and was detected both at maturity and mid-development (Fig. 9, B to D) (Supplementary Fig. S5). Plant to plant variation was ruled out as the cause of the observed structural difference by comparing segregating sibling kernels grown on the same ear (Fig. 9C).

Deletion of ISA2 also affected the chain length distribution in the phytoglycogen that accumulates in *su1-Ref* mutants (Supplementary Fig. S6). The magnitude of the change was less for phytoglycogen than for amylopectin, and the degree of variability between biological replicates was greater. Nonetheless, a distinctive phytoglycogen chemotype cosegregated with the *su1-Ref*, *isa2-339* double homozygous genotype. Like the remnant amylopectin, the relative abundance of the shortest linear chains was elevated by addition of *isa2-339* to the *su1-Ref* background. The pattern was reproducible over generations and in sibling kernels from the same ear, and was observed in phytoglycogen from mature or mid-development kernels (Supplementary Fig. S6).

The same effect of ISA2 deletion on amylopectin chain length distribution was also observed in homozygous *su1-Bn2* and *su1-st* backgrounds. As with *su1-Ref*, ISA2 loss in either *su1-Bn2* or *su1-st* homozygous backgrounds affects structure to increase the abundance of the shortest linear chains in the molecule (Supplementary Fig. S7). These data demonstrate a reproducible genetic effect of ISA2 deletion on the structure of α-polyglucans that accumulate in the absence of ISA1 catalytic activity, including hydrosoluble polymers and those that assemble into starch granules.

### Effects of eliminating ISA2 in a *su1-ref/+* heterozygous background

**Figure 10.**
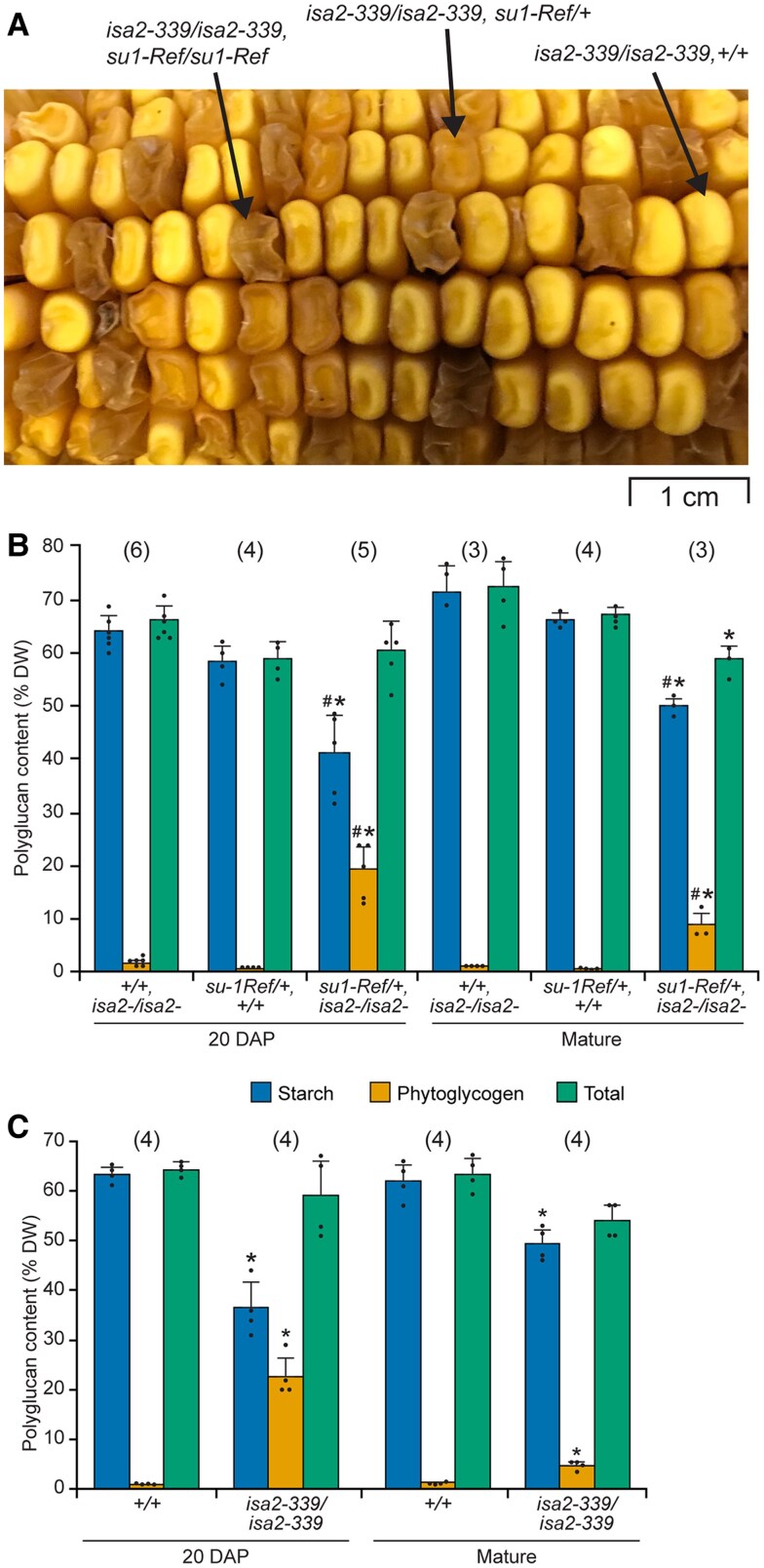
Visual phenotypes and chemotypes caused by loss of ISA2 in kernels containing nonmutant alleles at the *su1* locus. **A)** Mature ear from a cross between *isa2-339/isa2-339*, *su1-Ref/+* plants. Genotypes were determined from genomic DNA of individual kernels. A section of this image is also shown in Fig. 4A. **B)** Starch and phytoglycogen content in the W64A inbred background. Values are average ± SD as a percentage of dry weight. The number of biological replicate kernels analyzed is shown in parentheses. Black dots indicate values from individual biological replicate kernels. The *su1-Ref/+, isa2*-*339/isa2-339* kernels and *+/+, isa2-339/isa2-339* kernels are siblings from the same ear, from a cross such as that shown in Panel **(A)**. The *su1-Ref/+, +/+* kernels are from a separate ear. Pound signs (#) indicate significant difference from the corresponding *+/+, isa2-339/isa2-339* line. Asterisks (*) indicate significant difference from the corresponding *su1-Ref/+, +/+* line. Significance is noted at *P* value < 0.006 (two-tailed Student's *t*-test). **C)** Starch and phytoglycogen content in the Ia453Su1 + inbred background. “+” indicates the nonmutant allele of the *isa2* locus. Values are average ± SD as a percentage of dry weight. The number of biological replicate kernels analyzed is shown in parentheses. Black dots indicate values from individual biological replicate kernels. Asterisks (*) indicate significant difference from the corresponding nonmutant line (two-tailed Student's *t*-test, *P* value ≤ 0.002).

Congenic lines were established that compare presence or absence of ISA2 in endosperm heterozygous for the nonmutant allele *Su1* (denoted as “*+*”) and the mutant allele *su1-Ref.* These individuals express both nonmutant ISA1 (ISA1-WT) and ISA1-W578R for assembly into the various ISA complexes. In otherwise wild type backgrounds *su1-Ref/+* heterozygotes exhibit a normal phenotype in terms of kernel appearance and starch content (Figs. 4D and  10), and are devoid of phytoglycogen. Thus, *su1-Ref* is fully recessive to the nonmutant allele *Su1*.

Crosses between *isa2-339/+, su1-Ref/+* plants yielded ears on which approximately one-eighth of the kernels exhibited a unique visual phenotype distinguishable from wild type or *su1-Ref* homozygous individuals. These were provisionally assigned the genotype of *isa2-339/isa2-339, su1-Ref/+*, based on Mendelian segregation ratios. Plants grown from the intermediate phenotype kernels were crossed. In the next generation, the intermediate phenotype was evident in approximately one-half of the mature kernels (Figs. 4A and 10A), as predicted from the provisional genotype. Alleles at the *isa2* and *su1* loci in these kernels were identified by genomic PCR or genomic sequencing, respectively. Kernels with the intermediate phenotype invariably exhibited the expected genotype of *isa2-339/isa2-339, su1-Ref/+*.

Starch and phytoglycogen content in endosperms in the *su1-Ref/+* background, as well as the nonmutant background (*+/+*), could then be compared between siblings either normal for ISA2 or lacking ISA2, with all lines congenic in the W64A background. Kernels of the genotype *isa2-339/isa2-339, +/+* have normal visual appearance and starch content, and lack phytoglycogen (Fig. 10, A and B). In contrast, in *su1-Ref/+* heterozygous lines loss of ISA2 conditioned appearance of phytoglycogen and the characteristic intermediate kernel phenotype. Phytoglycogen content in *isa2-339/isa2-339, su1-Ref/+* endosperm was less than in double homozygous mutant tissue (*isa2-339/isa2-339, su1-Ref/su1-Ref*) (Fig. 5, B and D), consistent with the intermediate kernel phenotype. ISA2 deletion in *su1-Ref/+* kernels also affected the starch:phytoglycogen ratio in immature kernels (Fig. 10B). These results from near-isogenic W64A lines reiterate a previous study using recombinant inbreds that varied *su1-Ref* endosperm gene dosage in the *isa2-339* homozygous background (De Vries and Tracy 2016). In that study appreciable phytoglycogen content was also detected in *isa2-339/isa2-339, su1-Ref/+* endosperm. These data indicate that ISA complexes assembled from a mixture of ISA1-WT and ISA1-W578R can function to produce a normal chemotype if ISA2 is present, but not if ISA2 is absent.

### Elimination of ISA2 in a sweet corn inbred background

Ia453Su1 + is an inbred originally derived from several *su1-Ref* sweet corn land races that subsequently was converted by backcrossing to a nonmutant *Su1* derivative (De Vries and Tracy 2016). The sweet corn genetic background of Ia453Su1 + is phylogenetically distinct from dent maize lines and thus likely contains substantial allelic diversity compared with inbred W64A (Hu et al. 2021). Presence of a nonmutant allele at the *su1* locus in Ia453Su1 + was confirmed by observation of active ISA1 homodimer and ISA1/ISA2 heteromultimer in nondenaturing in-gel assays (Fig. 6B) and by genomic sequence analysis. The null allele *isa2-339* was introgressed into Ia453Su1+ (Supplementary Fig. S3E). Mature kernels heterozygous for *isa2-339* do not exhibit any abnormality in the visual phenotype, i.e. *isa2-339* is fully recessive to the nonmutant allele. Sibling crosses between *isa2-339/+* heterozygotes resulted in ∼25% shrunken and translucent kernels (Fig. 4E). This phenotype bred true over multiple generations. PCR genotyping invariably showed mutant kernels in the segregating populations to be *isa2-339* homozygotes. Nucleotide sequence analysis confirmed all segregants were homozygous for the nonmutant allele *Su1*, ruling out possible contaminating *su1-*mutant pollen.

The kernel phenotype of the ISA2-null line in the Ia453Su1 + background indicated presence of phytoglycogen, despite presence of functional ISA1. Phytoglycogen was detected in *isa2-339* homozygous mutant endosperm both at mid-development and maturity, along with decreased starch content (Fig. 10C). This is contrary to the *isa2-339* conversion of otherwise nonmutant W64A that does not display an abnormal kernel phenotype nor accumulate phytoglycogen (Figs. 4E and 10B). Presumably, uncharacterized allelic diversity between the genomes of Ia453Su1 + and W64A accounts for the varying effects of *isa2-339* between inbred genotypes.

### Molecular modeling of ISA complexes

**Figure 11.**
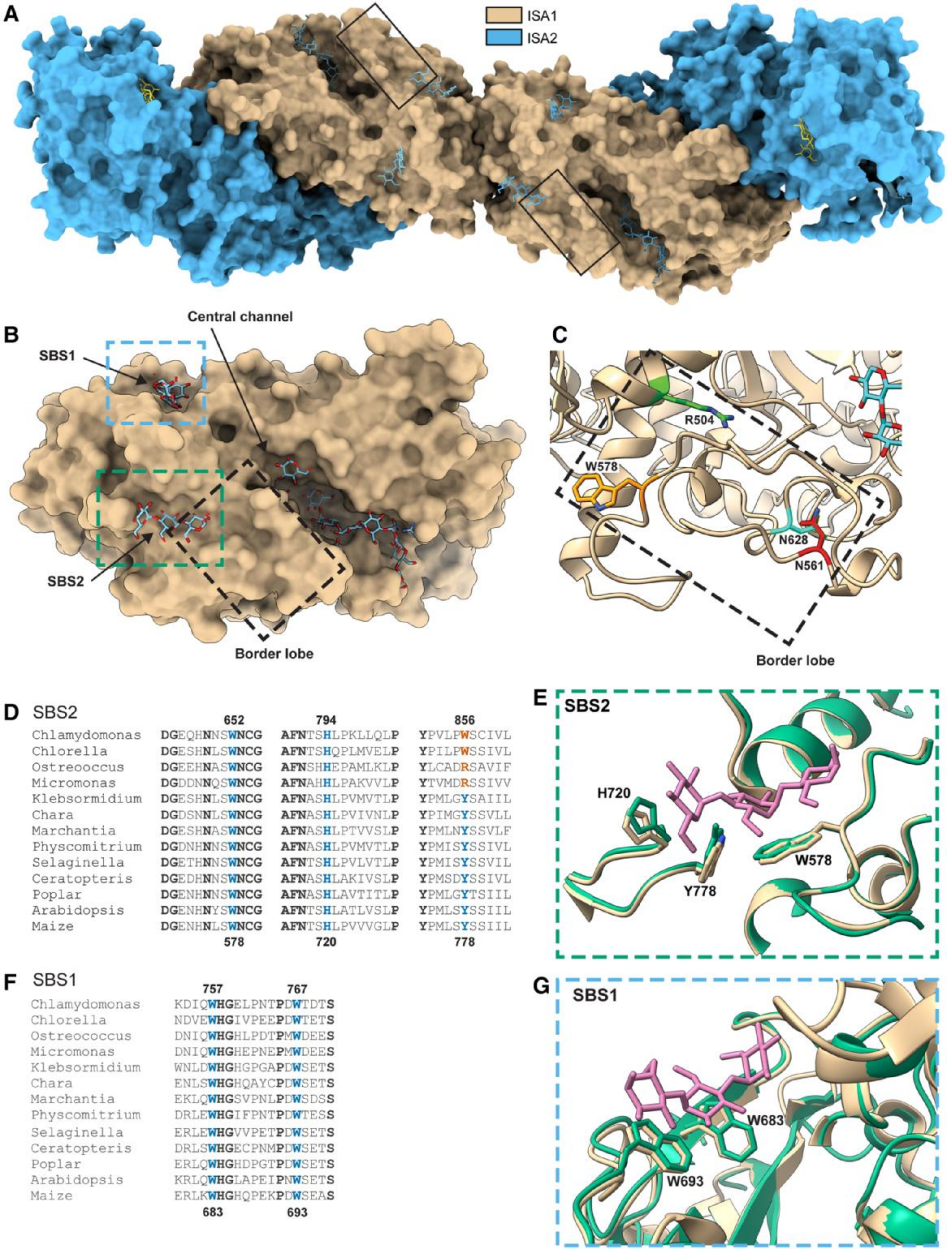
Maize ISA1/ISA2 structural modeling. Additional details are in Supplementary Fig. S9. Structure models were determined using AlphaFold 3. Bound glucans were positioned based on the structure of Chlamydomonas ISA1 containing bound maltoheptaose (Protein Data Bank identifier 4OKD). **A)** Surface model of the maize ISA1/ISA2 heterotetramer. Glucans bound to ISA2 are colored yellow and glucans bound to ISA1 are colored sky blue. Boxes indicate the border lobe in ISA1. **B)** Surface model of maize ISA1. Dotted line boxes are expanded in panels **(C), (E)**, and **(G)** according to color. **C)** Location of *su1-*mutation sites within the border lobe. **D)** ISA1 primary sequence conservation surrounding the glucan contact sites in secondary binding site 2 (SBS2). Blue text indicates glucan contact residues and vermillion text indicates variation from the conserved sequence. Bold black text indicates residues conserved in all of the analyzed proteins. Residue numbers are indicated for maize and Chlamydomonas ISA1. GenBank identifiers of the analyzed sequences are: Chlamydomonas, AAP85534: Chlorella, KAL4541998; Ostreococcus, OUS46015; Micromonas, XP_003059993; Klebsormidium, GAQ88556; Chara, GBG67506; Marchantia; PTQ45100; Physcomitrium, XP_024394371; Selaginella, XP_024531535; Ceratopteris, KAH7288477; Poplar, XP_034893113; Arabidopsis, AEC09752; maize, XP_008678357. **E)** Structural conservation of SBS2. Tan color indicates Chlamydomonas ISA1, bluish green color indicates maize ISA1, and reddish purple color indicates bound glucan. Residues that directly contact bound carbohydrate are numbered according to the maize ISA1 sequence. **F)** ISA1 primary sequence conservation surrounding the glucan contact sites in secondary binding site 1 (SBS1). Details are as in Panel **(D)**. **G)** Structural conservation of SBS1. Details are as in Panel **(E)**.

Structural modeling of the ISA1/ISA2 heterotetrameric complex was used to investigate how the *su1-*mutations (Table 1) affect catalytic activity and contribute to variation in the starch/phytoglycogen ratio. A heterotetramer with 2 subunits each of ISA1 and ISA2 was modeled using AlphaFold3 (Abramson et al. 2024). The prediction closely matches the structure of a rice ISA1/ISA1/ISA2 heterotrimer determined by cryoEM (Fan et al. 2025). The positions of the mutated residues were then mapped onto the maize heterotetramer model. Previous analysis had shown that the residues changed by *su1-Ref* (W578), *su1-NC* (R504), or *su1-SW* (N561) are located relatively close to each other in the ISA1 monomer (Tracy et al. 2006). Mapping the *su1-Bn2* mutation site, N628, showed that not only is it located in the same region of the protein, but it directly contacts N561 (Fig. 11, A to C).

The area of ISA1 with the 4 mutation site is located in a discrete lobe on the protein surface referred to as the “border lobe.” The border lobe is not involved in the interface between the ISA1 subunits that forms the ISA1 homodimer, nor is it near the interface between ISA1 and ISA2 (Fig. 11A). Thus, the mutations are unlikely to affect complex formation. This prediction was verified for *su1-Ref* and *su1-Bn2* because the mutant and wild type ISA1 proteins exhibited the same elution volume in size exclusion chromatography (SEC) indicative of a high molecular weight complex (Supplementary Fig. S8). ISA2 eluted in the same SEC fractions as ISA1 in *su1-Ref*, *su1-Bn2*, and nonmutant endosperm, indicative of heteromultimeric complex assembly. These data also indicate the mutations in ISA1 do not affect the steady-state level of ISA2, as had been shown previously for *su1-Ref* and *su1-am* (Kubo et al. 2010).

Glucan binding sites were mapped onto the maize ISA1 AlphaFold3 model through structural alignment with the *Chlamydomonas* ISA1 crystal structure containing bound maltoheptaose (Sim et al. 2014). The *Chlamydomonas* protein was removed, leaving only maltoheptaose positioned in the maize ISA1 model (Fig. 11, A and B). This model showed that the border lobe forms one side of a clearly delineated α-polyglucan binding channel, referred to as the “central channel”, that traverses the active site (Fig. 11B). None of the 4 mutated residues directly participates in the surface of the central channel, and all of them are more than 12 Å from either the catalytic site or the nearest maltoheptaose atom bound in that location (Supplementary Fig. S9). Therefore, neither direct participation in the catalytic mechanism nor substrate binding at the catalytic site appears to be valid explanations for why the mutations prevent enzymatic activity.

In addition to the central channel, the *Chlamydomonas* ISA1 structure reveals 2 additional glucan binding sites designated as secondary binding site 1 (SBS1) and secondary binding site 2 SBS2 (Sim et al. 2014) (Fig. 11B). The residues that directly contact glucan in both of those sites are highly conserved throughout the Chloroplastida lineage (Fig. 11, D and F), and structural overlay shows these side chains to be essentially superimposable between *Chlamydomonas* and maize ISA1 (Fig. 11, E and G). Thus, SBS1 and SBS2 likely contribute to ISA1 function. Notably, one side of the border lobe, separate from the part facing the central channel, contributes to SBS2 (Fig. 11B). Further, the site of the *su1-Ref* mutation, W578, is one of the residues in SBS2 that directly contacts the bound glucan (Fig. 11E). Thus, *su1-Ref* likely affects ISA1 function by interfering with α-polyglucan binding at SBS2. The other 3 mutation sites in the border lobe do not directly contact the bound glucan, but may affect SBS2 structure or how glucan bound within that site interacts with the neighboring surface on ISA1.

**Figure 12.**
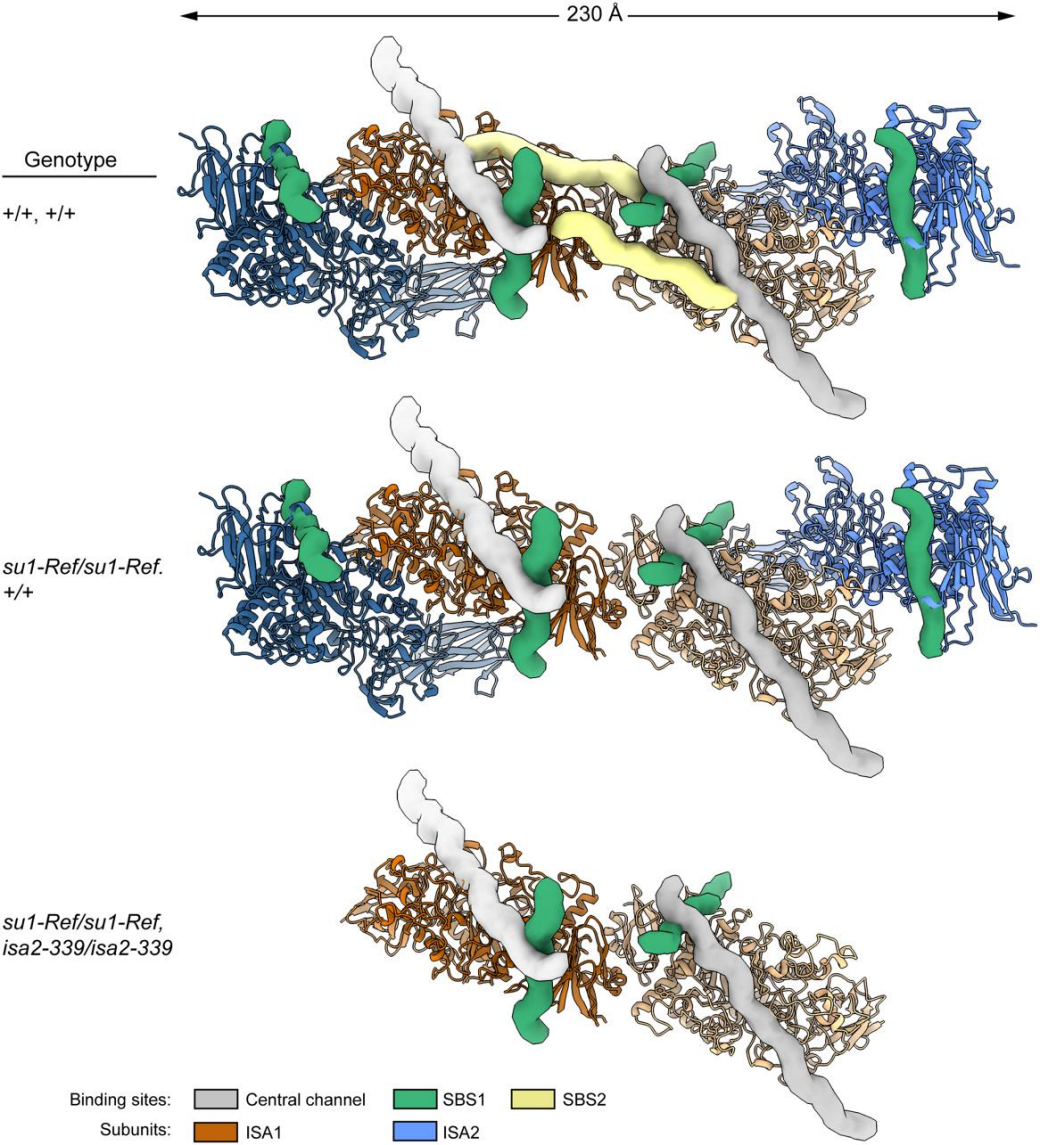
Model of the ISA1/ISA2 heterotetramer bound to linear chains within a branched α-polyglucan. Protein structure was modeled using AlphaFold3. Tubes model glucan polymers as described in Materials and methods. The glucan chains bound to SBS1 and SBS2 are the approximate diameter and length of an α–polyglucan containing 14 glucose units, and the chain bound to central channel models an α–polyglucan with 28 glucose units. Glucans are modeled in an extended conformation extruded from the defined binding sites (Fig. 11, A and B) that does not account for inherent flexibility of the polymer when not directly contacting the protein surface. The models for *su1-Ref* mutants assume that binding at SBS1, SBS2, and the central channel occurs independently of one another.

The heterotetramer model provides insight into why loss of ISA2 exacerbates the level of phytoglycogen accumulation in the absence of any catalytic activity. Eight α-polyglucan binding sites are evident in the heterotetramer (Fig. 12). Each ISA1 subunit contributes SBS1, SBS2, and the central channel, whereas each ISA2 likely contributes only SBS1. The 2 Trp residues that directly contact glucan in SBS1 of ISA1 are present also in ISA2, and this motif is broadly conserved in ISA2 from diverse Chloroplastida species (Supplementary Fig. S10). In contrast, the SBS2 sequence of ISA1 is not found in ISA2. Also, AlphaFold3 modeling of ISA2 predicts a loop structure within the central channel that would preclude glucan binding. These predictions are supported by glucan occupancy at all 3 positions in ISA1, and only SBS1 in ISA2, in the cryoEM structure of the rice ISA1/ISA1/ISA2 heterotrimer (Fan et al. 2025). Only a subset of the 8 possible glucan binding sites will be available in ISA complexes when mutations in either ISA1 or ISA2 are combined (Fig. 12). Variation in this parameter may explain why loss of ISA2 causes increased phytoglycogen content in the absence of ISA catalytical activity, i.e. in *su1-, isa2-*double mutants compared with *su1-*single mutants.

Molecular modeling of glucan binding sites in the maize heterotetramer does not include a location in the CBM48 module of either ISA1 or ISA2. Primary sequence alignments showed that several generally conserved residues within CBM48 vary in that module specifically in the GH13_11 subfamily (Janecek et al. 2011). Several of these conserved residues directly contact bound glucans in CBM48-containing proteins such as SEX4 (Meekins et al. 2014). Structural alignment between the CBM48 modules of SEX4 and ISA1 indicated the latter lacks the glucan binding site (Supplementary Fig. S11). This is supported by absence of bound maltoheptaose to CBM48 in the known structure of the Chlamydomonas ISA1 homodimer or the rice ISA1/ISA2 heterotetramer (Sim et al. 2014; Fan et al. 2025). Thus, all 8 glucan binding sites modeled in the maize heterotetramer are independent of the CBM48 domain.

## Discussion

### Variation in starch/phytoglycogen distribution in the absence of ISA enzymatic activity

The ISA1/ISA2 complexes of maize endosperm were shown to have a function that does not involve α-(1→6)-glucosidase enzymatic activity. This is evident from the observation that multiple noncatalytic missense variants of ISA1 generate substantially more starch than the amount in tissue devoid of ISA1 protein. An illustrative example is ISA1-N628K encoded by *su1-Bn2*. This mutation results in mature endosperm that contains ∼55% starch as a percentage of dry weight (Fig. 5C), whereas mutants devoid of ISA1 protein accumulate only 10% starch (Dinges et al. 2003b; Kubo et al. 2010). ISA1-N628K protein accumulates to the same level as wild type ISA1 (Fig. 6A), consistent with an active function that influences starch content. The possible explanation that unspecified allelic variation explains the different starch levels was ruled out by extensive backcrossing of the mutant alleles into a consistent genetic background. The noncatalytic missense mutants all accumulate less starch than nonmutant endosperm. Thus, the overall role of ISA complexes in starch biosynthesis is provided by a combination of catalytic and noncatalytic functions.

Further evidence for a noncatalytic role of the ISA complexes comes from comparing the effects of noncatalytic ISA1 missense variants in the presence or absence of ISA2. In every instance removal of ISA2 in lines containing noncatalytic ISA1 decreased starch accumulation and increased phytoglycogen content compared with the effect of the ISA1 mutation by itself (Fig. 5). In addition, removal of ISA2 also substantially changed the structure of the remaining amylopectin and the accumulated soluble polymer, compared with the congenic *su1-*single mutant. Considering that ISA2 is noncatalytic, its presence or absence is unlikely to alter the enzymatic capacity of ISA1 variants that by themselves are also noncatalytic. This provides another instance in which the extent of starch formation varies independently of the capacity to hydrolyze branch linkages. The results also indicate that ISA2 is involved in the noncatalytic function of the complexes.

An alternative explanation for the observed chemotypic variation may be that the determining factor is α-(1→6)-glucosidase activity below the level of detection. Potentially the various alleles could each reduce enzyme function to different extents, even though such remnant activity is too low to be detected by in-gel enzyme assays or activity assays with recombinant proteins. This is unlikely because the critical amount of activity necessary for ISA function to control phytoglycogen content would have to be far below the observed capacity in nonmutant tissue. Also, the already undetectable level of enzymatic activity would have to be reduced even further by removal of ISA2. Although such a scenario cannot be ruled out because it posits activities that cannot be observed, the most likely explanation is an aspect of ISA complex function that is independent of catalytic capacity.

### Potential ISA functions independent of catalysis

Starch polymers are synthesized initially in the soluble phase, and then subsequently crystallize as they assemble into insoluble granules. Several explanations can be envisioned for how ISA complexes could affect this transition independent of the ability to hydrolyze branch linkages. One possibility is that the ISA complexes mask certain areas of precursor polymers from other carbohydrate-active enzymes. For example, binding of an ISA complex to soluble α-polyglucan precursor could restrict that region of the polymer from being used as substrate of SBE. Nonmutant ISA could thereby create unbranched regions both through its hydrolytic activity and by providing a physical impediment to other enzymes, and this latter function would remain in the mutant variants.

Another possible explanation is that ISA complexes attract other enzymes to the precursor polymer as it is being synthesized. There is ample surface area potentially exposed to solute on both the ISA1 and ISA2 subunits of the complexes, and these could form binding surfaces for attachment of other polypeptides. The border lobe in ISA1, where 4 missense mutation sites are located, is part of the exposed surface. Genetic interactions between ISA1, ISA2, and other carbohydrate-active enzymes have implicated both SSIIIa and the maize protein ZPU1 as potential binding partners (Mangelsdorf 1947; Dinges et al. 2003a, 2003b; Lin et al. 2012). ZPU1 has been shown to interact directly with ISA1 (Boehlein et al. 2025). This is notable because ZPU1 is another α-(1→6)*-*glucosidase of the GH13 family, and as such is structurally similar to ISA including multiple glucan binding sites. Other candidates for interaction with the ISA1/ISA2 complexes are homologs of the LESV2 and PTST2 proteins of Arabidopsis (Seung et al. 2015). These are noncatalytic proteins known to bring target enzymes to starch granules. The rice homologs, FLO6 and FLO9, respectively, have been reported to bind to ISA1 (Yan et al. 2024). Further analyses will be required to unravel the details of which other proteins might interact with the ISA complexes.

A third possible explanation is that ISA complexes provide nucleation sites that stimulate α-polyglucan crystallization. Molecular modeling indicated that an ISA1/ISA2 heterotetramer contains 8 separate locations where a glucan chain can bind, 3 in each ISA1 subunit, and one in each ISA2 subunit (Fig. 12). All of these appear to be conserved throughout the Chloroplastida lineage, and direct structural analyses revealed that maltoheptaose can bind to all these sites (Sim et al. 2014; Fan et al. 2025). Binding of multiple glucan chains on a single ISA complex may bring them into proximity and thus increase the propensity for double helices to form. In this way, the ISA1/ISA2 complexes could act as chaperones for assembly of a complete three-dimensional structure of crystalline α-polyglucan.

These hypothetical explanations for how ISA1/ISA2 complexes may function in the absence of catalytic activity are not mutually exclusive. For example, stimulating crystallization by a proximity effect could occur at the same time that ISA attracts additional carbohydrate-active enzymes to the same location on the polymer. In the context of nonmutant proteins with normal catalytic capacity, some chains could be removed from the precursor while other chains are induced to form double helices and thus initiate crystallization. A further consideration is that in some nonmutant tissues multiple forms of the complexes exist. Maize endosperm, for example, contains an ISA1 homodimer, and at least 2 electrophoretic mobility forms of ISA1/ISA2 heteromultimer, all of which possess catalytic activity (Kubo et al. 2010; Trimble et al. 2016). Each of these assembly forms are expected to vary regarding the assortment of glucan binding sites they possess.

Structural features that cannot be addressed by the models shown here are likely to be critical for how ISA complexes function. These include the degree of interdependency of glucan binding at each of the sites in any of the complexes, and the way that glucan chains within a branched polymer are arranged between the fixed glucan binding sites observed by static methods, i.e. X-ray crystallography or cryoEM.

### Effects of mutations in ISA1 and ISA2

Molecular modeling provided insight into (i) how the missense changes in ISA1 cause loss of catalytic function, (ii) why the substitutions vary relative to each other regarding resultant starch content even though all of them are noncatalytic, and (iii) how ISA2 contributes to determining starch formation. The ISA1 border lobe clearly provides a function that is necessary for catalysis. This location does not form any part of the interfaces between subunits in the heterotetramer, nor does it appear to be directly involved in the catalytic mechanism, however, it could affect glucan binding. The *su1-Ref* substitution directly affects the SBS2 glucan binding site, and replacement of a tryptophan residue that normally directly contacts glucan with an arginine likely disrupts binding at that site. Thus, correct glucan binding at SBS2 appears to be required for ISA1 catalytic activity. A possible explanation is that SBS2 binds the branch that is to be released by hydrolysis, while the central channel binds the linear chain to which that branch is attached by its reducing end α-(1→6) glycoside bond. According to this hypothesis, binding at both locations would be necessary for catalysis. The other border lobe mutations, while not directly involved in glucan binding at SBS2, could affect the coordination between that site and binding within the central channel. Consistent with this hypothesis, the residue affected by *su1-NC*, R504 lies directly between SBS2 and the active site (Fig. 11C).

Differences in the interaction between ISA1/ISA2 complexes and branched α-polyglucan precursors likely explains why missense mutants vary in their noncatalytic functions as evidenced by specific levels of starch accumulation. Presence or absence of ISA2 also is likely to affect interaction between the complexes and precursor polymers, again changing the starch level in the absence of catalytic activity. Variable glucan binding specificities are proposed to result from different assortments of the possible glucan binding sites in the ISA complexes. The heterotetramer model is presented in the context of α-polyglucan chains with a degree of polymerization up to DP28, which is likely considering the nature of amylopectin, phytoglycogen, and their precursors (Fig. 12). The glucan binding sites are distributed along an extended axis with intervening distances that could accommodate numerous monosaccharide monomers within a polymer. Thus, it is likely that ISA1/ISA2 complexes can bind to regions of branched α-polyglucan that are widely separated within the primary structure of the polymer.

According to this model, association of glucan chains with the ISA complex will be changed by amino acid substitution in the *su1-*mutants and/or by loss of ISA2. As noted, the *su1-Ref* mutation likely prevents glucan binding at SBS2, so complexes containing that mutant version of ISA1 will have only 6 binding sites available rather than 8 in the wild type complex (Fig. 12). Further, removing ISA2 from the *su1-Ref* mutant complex will reduce the available binding sites to only 4. Such assortment of binding sites is proposed to influence the affinity of ISA1/ISA2 complexes for precursor polymers, and this in turn would affect the extent to which the proposed noncatalytic functions can operate. Beyond a general affinity, assortment of binding sites could affect ISA complex binding to specific regions of the precursor polymer that likely vary regarding the length, density, and/or relative positioning of branched chains. This in turn would influence where the noncatalytic functions are executed and thus result in differences in the organization of crystalline regions and eventually in distinct starch contents. Regarding specific starch levels conditioned by various substitutions in the border lobe, binding affinity at SBS2 could be affected differently for each change. This would result in further variation in the interaction between the ISA complexes and the precursor polymers, and hence in distinct starch/phytoglycogen ratios.

The *su1-am* allele affects a residue elsewhere in ISA1 relative to the border lobe. This allele has complex genetic effects. Specifically, *su1-am* has no effect when homozygous in an otherwise nonmutant background, but conditions phytoglycogen accumulation if ISA2 or SSIIIa is missing (Mangelsdorf 1947; Kubo et al. 2010). ISA1-R308I does not form active ISA1/ISA1 homodimer, but is able to form active complexes containing ISA2 (Kubo et al. 2010). How changes at R308 could result in genetic interactions with multiple effectors, considering its location in the interior of ISA1, remains to be determined.

The *su1-st* allele presents a special case. ISA1 appears to be completely absent from *su1-st* homozygous mutant endosperm, i.e. no detectable protein accumulates, so the resultant phenotype is expected to be the same as that of the insertion allele *su1-4582* that produces no protein. This is not the case, however, as the *su1-st* chemotypes, and the genetic effects of this mutation, are clearly distinct from those of *su1-4582*. A possible explanation is that the anti-ISA1 IgG fraction used here, which was raised against a peptide sequence located on the ISA1 surface, does not bind to the ISA1 variant produced by *su1-st*. The *su1-st* allele is caused by an insertion in exon 10 that results in abnormal splice site utilization during pre-mRNA processing (Dinges et al. 2001). Several transcript variants are produced, one of which encodes a 6 residue insertion in ISA1 and another that encodes a 10 residue deletion variant. Whether either of these polypeptides accumulate is unknown, although some protein product is expected given the genetic effects of the allele.

### Evolutionary selection of ISA2

Missense changes that prevent catalytic function of an enzyme, such as have occurred in ISA2, are expected a priori to generate pseudogenes that are no longer subject to selection and can be lost from the population. ISA2, however, was subjected to continuous evolutionary selection starting from a common ancestor of the Chloroplastida lineage. The observation that ISA2 underwent more rapid sequence divergence than ISA1 or ISA3 may be explained by its selection not being dependent on catalytic function. In addition to losing catalytic function, the progenitor ISA2 incorporated the N terminal β-sandwich domain that is present only in this single branch of the broad GH13_11 subfamily. Retention of this domain throughout subsequent evolution suggests it also plays a role in the selected function of ISA2.

The data presented here suggest a possible selective pressure that may explain maintenance of ISA2, which is the ability to modulate the starch/phytoglycogen ratio within plastids. Although extant species produce only starch, progenitors are expected to have accumulated both glycogen and starch. Primary endosymbiosis integrated the genomes of 2 species that both produced only glycogen, and this transitioned into a starch based system. Such a drastic change, involving the integration of enzymes from the different genomes, is highly unlikely to have occurred instantaneously. The alternative is that intermediate species produced both soluble and semicrystalline α-polyglucans. This applies also during transition of starch metabolism into the chloroplast, moving from the cytoplasm where it was originally established. This required the attachment of plastid targeting peptide coding sequences to multiple open reading frames in the nuclear genome, necessary to deliver SS, SBE, and other proteins involved in granule initiation and catabolism, including the ISA proteins. This stage of evolution is when the 3 ISA paralogs appeared (Deschamps et al. 2008).

A reasonable scenario is that soluble α-polyglucans were synthesized as biosynthetic factors including SS and SBE arrived in the plastid stroma. This again converted to a starch-based system, likely with the participation of ISA1, while ISA3 retained the catabolic function necessary for starch utilization. As this transition took place, the presence of ISA2 could have provided the ability to adjust metabolism to integrate the changes taking place as photosynthetic efficiency increased. Glycogen and starch both offer advantages for hexose storage, for example rapid release from the soluble form but less storage capacity, compared with much greater storage capacity in the insoluble form but slower release of glucose when catabolism is required. Adjusting the ratio of the soluble and insoluble forms of α-polyglucan could have been advantageous during the numerous evolutionary steps that led to the spread of land plants. This may explain the persistence of ISA2, and why in some instances it is dispensable for normal phenotypes whereas in others it is required. Notably, ISA2 can adjust the starch/phytoglycogen ratio even in conditions when functional ISA1 is present (Fig. 10).

The concept of adjustment in the starch/phytoglycogen ratio as a basis of selection has precedent in the creation of sweetcorn lines. Mutation of ISA1 repeatedly caused a change from starch-only metabolism to mixtures of starch and phytoglycogen. Here the selection was not evolutionary fitness, but instead was a kernel phenotype that makes sweetcorn a desirable vegetable. This arises from phytoglycogen in immature kernels that imparts consumer-preferred texture, and elevated sucrose content.

### Potential application of variability created by ISA2 deletion

Mutations of the *isa2-*locus are potentially useful in sweetcorn breeding. The effects of ISA2 deletion have been tested over multiple years, at different stages of development, and on sibling kernels developing on the same ear, all with essentially the same results. Further, ISA2 was eliminated independently in 4 different *su1-*mutant backgrounds, again with a uniform effect of increasing phytoglycogen content. Thus, the effects of *isa2-339* are likely to be highly reproducible. Comparison among the various allele combinations at the *su1* and *isa2* loci reveals a great degree of variation in the division between starch and phytoglycogen both at the eating stage of 20 DAP and at maturity (Fig. 5). Elevated phytoglycogen content may be a desirable trait in some contexts, whereas in others increased levels of starch might improve some aspects of crop performance. Correlation between phytoglycogen content and sucrose level is strong and sugar levels are likely to remain elevated in lines based on these *isa1-* and/or *isa2-*alleles.

ISA2 mutation also provides the ability to generate phytoglycogen without involving *su1-Ref*. For example, the starch/phytoglycogen division in *su1-Bn2*, *isa2-339* double homozygotes is similar to that conditioned by *su1-Ref* as a single mutant (Fig. 5). This may be advantageous in the long term from the standpoint of crop security. The *su1-Bn2* allele arose in an entirely different haplotype than did 3 alleles found in sweetcorn accession lines, namely *su1-Ref*, *su1-NC*, or *su1-SW*. Sweet corns including *isa2-339* and *su1-Bn2*, therefore, would contain a different set of allelic variation than *su1-Ref* lines in the vicinity of the *su1* locus on chromosome 4S as well as throughout the genome. Another possible advantage of using *isa2-339* coupled with alternative alleles of the *su1* locus in sweetcorn breeding might be the ability to modulate starch content in phytoglycogen-producing lines to improve germination rates or other seed-related traits.

## Materials and methods

### Maize lines

#### Near-isogenic maize (*Zea mays* L.) lines involving *su1-Ref* and *isa2-339*

Maize was propagated in field conditions at ISU Curtiss Farm, Ames, IA, during successive summer nurseries. Near-isogenic lines were generated by repeated backcrosses in the W64A inbred background starting from *isa2-339* or *su1-Ref* single mutant parents. Homozygous lines were repeatedly outcrossed to W64A standard, and then F1 plants were crossed in the next generation to regenerate mutant seed. Progeny kernel genotypes were assigned by visual phenotype for *su1-Ref* and PCR genotyping for *isa2-339*.

Plant numbers that follow refer to the pedigree shown in Supplementary Fig. S3A. In the 2017 summer nursery in Ames, IA, an *isa2-339/+, su1-Ref/+* F1 plant (17*-*5161*-*8) was crossed to the *isa2-339/isa2-339* parent line. Approximately 1/4th of the progeny on the resultant ear displayed a moderately shrunken and wrinkled visible kernel phenotype. Plants derived from such seeds in the 2018 nursery were identified by PCR analysis as *isa2-339* homozygotes (plant 18*-*6298*-*4 and siblings). Crossing these plants yielded ears segregating for normal kernels, severely shriveled and translucent kernels typical of *su1-Ref* homozygotes, and the moderately wrinkled and translucent phenotype of the parent seeds, defined as “slight sugary” (Fig. 4A) (Fig. 10A). Genomic sequence analysis of embryo DNA invariably identified the slight sugary kernels as *su1-Ref/+* heterozygotes and the severe sugary kernels as *su1-Ref* homozygotes.

Plants grown from slight sugary kernels were crossed to each other in the 2019 nursery (plants 19*-*7303*-*1,3). Severe sugary segregant seed derived from these crosses were propagated in 2020 and 2021, yielding populations of mature *isa2-339, su1-Ref* double homozygous kernels subjected to further analysis (ears from plants 20*-*8342-4, 21-8191-3, and 21-8192-2). Genotype determination by PCR and genomic sequencing of embryo DNA from the 2021 kernels confirmed the lines as *isa2-339, su1-Ref* double homozygotes. Mature double mutant kernels were also obtained from a segregating ear grown in 2019 (plant 19-7303-1). Double mutant immature kernels harvested 20 DAP were obtained from segregating ears (plants 18-6296-9, 20*-*8343-4, and 21*-*8194*-*5). In these instances, endosperm genotype was determined by sequencing genomic DNA from the corresponding embryo. Double mutant endosperms were compared with *isa2-339* or *su1-Ref* single mutants from separate plants grown and harvested in the same conditions.

Another ear from this pedigree provided mature *isa2-339, su1-Ref* double mutants and *su1-Ref* single mutant kernels that developed on the same ear rather than separate plants. A double heterozygous F1 plant (17-5161-7) was crossed in 2017 to the *su1-Ref* parent line. Homozygous *su1-Ref* progeny kernels, identified by the severe sugary phenotype, were planted in 2018 and the *isa2* genotypes of the resultant plants were assigned by PCR analysis. Crosses between *isa2-339/+, su1-Ref/su1-Ref* plants yielded ears bearing severe sugary kernels segregating for the *isa2* genotype (plant 18*-*6297*-*2). PCR analysis of embryo genomic DNA revealed the *isa2* genotypes of corresponding endosperms.

Endosperm of the genotype *isa2-339/isa2-339, su1-Ref/+* also was analyzed. Mature kernels of this genotype were identified by the slight sugary visible phenotype and collected from the segregating ear of plant 20-8343-1. Immature kernels were identified by genomic sequence analysis of embryo DNA from the segregating ear of plant 18-6296-9. Immature endosperms heterozygous for *su1-Ref* in an otherwise nonmutant background were obtained similarly from the ear of plant 19-7274-5.

#### Near-isogenic lines involving *su1-am*, *su1-st*, *su1-Bn*, and *isa2-339*

Double mutant pedigrees are shown in Supplementary Fig. S3, B to D beginning with homozygous mutants in the W64A inbred background. Double heterozygotes were self-pollinated to generate a strong sugary kernel phenotype distinct from the appearance conditioned by either single mutant alone (Fig. 4). These severely shriveled, translucent kernels were propagated, and the sugary phenotype bred true in successive generations. Double homozygous mutant seed were planted, and kernels were harvested 20 DAP or at maturity. Plants used for subsequent analyses are indicated in the pedigrees.

#### Introgression into a sweet corn inbred background

Inbred Ia453Su1 + is a nonmutant conversion line (*Su1/Su1*) derived from several sweet corn land races homozygous for *su1-Ref* (De Vries and Tracy 2016). Homozygous *isa2-339* in W64A was crossed to Ia453Su1+, then in successive generations heterozygotes were again crossed to Ia453Su1 + as the recurrent parent (Supplementary Fig. S3E). Presence of *isa2-339* was tracked through the pedigree by PCR analysis of plant DNA. After 5 backcross generations *isa2-339/+* heterozygotes were self-pollinated. Homozygous *isa2-339* mutant kernels were identified by a visual phenotype typical of phytoglycogen accumulating endosperms, and by PCR genotyping of embryo DNA.

#### Seed stocks

Seed stocks available for distribution are listed in Supplementary Table S1.

### Genotype determination at the *isa2* and *su1* loci

Frozen immature kernels were removed from storage at −80 °C, thawed at room temperature for 2 min, and then dissected to separate endosperm and embryo from maternal tissue. Endosperm was immediately refrozen in liquid N_2_ and returned to storage at −80 °C in individual tubes indexed to the embryo from the same kernel. Genomic DNA was extracted from the embryo as previously described (Lappe et al. 2018). PCR analysis identified either the wild type allele or the mutant allele *isa2-339*. The wild type fragment of 792 bp was amplified with genomic sequence primers Ak1f (5′*-*GGCT-GTTCGCAATGTTTGGC*-*3′) and Ak2r (5′*-*CAGGATCAAATGCTATGG-CTTCC*-*3′) (Kubo et al. 2010). These primers flank the transposon insertion and thus do not amplify *isa2-339*. The mutant fragment of 680 bp was amplified using Ak2r and Mu9242 (5′*-*AGAGAAGCCAACGCCA[A/T]CGCCTC[C/T]ATTTCGTC*-*3′) located within the terminal inverted repeat of the *Mutator* transposon insertion. PCR reactions in 25 µL final volume included 1× GoTaq Green buffer (Promega), 1.5 mm MgCl_2_, 5% DMSO, 0.2 mm each deoxyribonucleotide triphosphate, 1 µM each primer, 0.025 units GoTaq DNA polymerase (Promega), and 1 µL of genomic DNA. The thermocycling program was 95 °C, 2 min; [95 °C, 30 s; 58 °C, 30 s; 72 °C, 60 s] (×38); 72 °C, 5 min.

In other instances, the *isa2* genotype of mature endosperm tissue was determined. Mature kernels were imbibed in 1% lactic acid, 0.3% sodium metabisulfite solution, then pericarp was removed, and embryo was dissected from endosperm using a scalpel. Endosperm was saved at –20 °C in tubes indexed to the embryo from the same kernel. Extraction of genomic DNA and PCR conditions were the same for mature and immature embryo.

The *isa2* genotype was also determined from leaf tissue. Samples were taken with scissors at the 7 leaf stage from indexed plants, and lyophilized to dryness for 48 h. Leaf DNA was extracted as previously described (Saghai-Maroof et al. 1984) and analyzed by PCR using the same protocol described for embryo DNA.

The genotypes of individual kernels on ears segregating for *su1-Ref* were determined by genomic sequence analysis. A fragment of 440 bp was PCR amplified from embryo genomic DNA using primers su1set10F (5′-CAATTAGGTGGATTAGTG-3′) and su1set10R (5′-ATGAAAC-TCTAAAGTGCG-3′) (Supplementary Table S2). PCR conditions were as for *isa2* genotyping, except the buffer was 1× GoTaq clear buffer (Promega) and the annealing temperature was 52 °C. Amplified fragments were purified using the QIAquick PCR Purification Kit (Qiagen no. 28104). The nucleotide sequence of the amplified fragment was determined by the chain termination method using the same primers. Endosperms homozygous for *su1-Ref* were identified by presence of a C residue at the first position of codon 578, whereas the wild type allele has a T residue at that position and heterozygotes exhibited a mixture of T and C in that chromatogram peak.

### Carbohydrate extraction and quantification

Starch and phytoglycogen content in endosperm was determined as described previously (Dinges et al. 2001), with slight modifications. Phytoglycogen indicates the quantity of glucose units released from α-polyglucan polymer by amyloglucosidase digestion and so includes both relatively small maltooligosaccharides with low degrees of polymerization (DP) and relatively large phytoglycogen molecules with high DP. Immature endosperms dissected from kernels stored at –80 °C were ground in a mortar and pestle in a total volume of 2 mL of ice-cold H_2_O. A 0.5 mL sample of the total lysate was placed in a preweighed plastic weighing boat, evaporated to dryness for 24 h at 37 °C, then weighed to determine sample dry weight using a balance accurate to 0.1 mg. The remaining 1.5 mL of crude lysate was centrifuged for 10 min at 3,300 × *g* at 4 °C to separate insoluble glucans, i.e. the starch pellet, from soluble glucans in the supernatant. The supernatant was centrifuged in the same conditions a second time to remove residual starch granules, then transferred to a fresh 2 mL polypropylene tube and incubated for 20 min in a boiling water bath to inactivate enzymes in the cell extract.

The starch pellet from the original centrifugation was suspended in a total volume of 1 mL H_2_O, and a sample constituting 20% to 100% of the total was collected, with volumes chosen empirically for subsequent quantification steps to fall within the range of the standard curve. The starch sample was brought to 1 mL final volume in H_2_O, centrifuged as noted, then the pellet was washed twice further in 1 mL H_2_O and once in 1 mL 80% ethanol, with mechanical dispersion and vortexing to suspend the starch pellet during each washing step. Final washed starch pellets were air dried for 10 min, and then dissolved in 1 mL final volume of 90% DMSO during 20 min incubation in a boiling water bath, with intermittent vortexing.

Further steps were carried out at room temperature. For each assay a standard curve was generated from samples containing 0 to 9 mg glucose. Starch and phytoglycogen samples were diluted in H_2_O to fall within this range when quantified. Glucan content in both fractions was determined by first digesting with amyloglucosidase (AMG) (Megazyme E-AMGDF) to release glucose from all glucan polymers. Control samples lacking AMG were assayed to determine free glucose content. Free glucose was essentially zero in all starch samples, and only a few percent of the amount of glucose released from phytoglycogen. Assays were performed in a 96-well microtiter plate in a total volume of 50 μL reaction mix containing the sample, 50 mm sodium acetate, pH 4.8, 1 mm CaCl_2_, and 0.33 units of AMG. After incubation for 20 min at 50 °C, total glucose in each assay was determined by the glucose oxidase method (Megazyme K-GLUC). Each assay well received 200 μL of GOPOD reagent, and A_510_ was measured after incubation for 20 min at 50 °C. A_510_ was converted to glucose concentration by comparison to the standard curve calculated from assays on the same plate.

Carbohydrate content in mature endosperm was assayed by the same method. Mature kernels were first imbibed as noted in the previous section, and then the embryo was removed by dissection with a scalpel.

Sucrose was quantified similar to phytoglycogen except that AMG was replaced by β*-*fructosidase from a Sucrose/D-glucose assay kit (Megazyme no. K-SUCGL). This enzyme releases glucose from sucrose. Total glucose was then measured by the glucose oxidase method, and free glucose content was subtracted from this value to indicate sucrose content.

### Amylopectin and phytoglycogen glucan chain length distribution

Amylopectin and phytoglycogen chain length distributions were characterized by high performance anion exchange chromatography with pulsed amperometric detection (HPAEC-PAD) as previously described (Fontaine et al. 1993; Lappe et al. 2018). Apparent abundance of chains of each specific DP was calculated from the peak area as a percentage of the total peak area of all chain lengths included in the analysis.

### Sequencing of the *su1* locus

An 8.37 kb region of the *su1* locus containing the complete coding region was amplified by PCR from embryo genomic DNA in 12 overlapping fragments using the primer sets listed in Supplementary Table S2. Both strands of each fragment were sequenced by the chain termination method by the Iowa State University DNA Facility (https://dna.biotech.iastate.edu). This analysis was applied to plants homozygous for *su1-st*, *su1-Bn2*, *su1-Ref*, and *su1-am* after extensive introgression into the W64A inbred background.

### Phylogenetic analysis

Genes encoding ISA proteins were identified using several conserved regions of amino acid sequence as BLAST queries to search genome and transcriptome data without applying any filters or clade preference. The top 2000 blast hits with E value < 1^−10^ were selected and aligned using the MAFFT multiple sequence alignment program (Katoh and Standley 2013). This resulted in selection of a large diversity of prokaryotic genomes while the eukaryotes were restricted to Archaeplastida and dinoflagellates. BMGE software was used to select informative blocks within the multiple sequence alignment (Criscuolo and Gribaldo 2010), then preliminary trees were generated with Fasttree (Price et al. 2010) with a block size of 4 and the BLOSUM30 similarity matrix. A “dereplication” step was applied using TreeTrimmer (Maruyama et al. 2013) to reduce the size of monophyletic clades in order to lessen sequence redundancy. The same tools were used to perform alignment and block selection on this new set of sequences, and a tree was calculated with IQ-TREE and the ultrafast bootstrap method (Nguyen et al. 2015). This process was repeated iteratively and manual selection reduced the diversity to obtain the final set of sequences analyzed. The final tree was obtained using the same tools for alignment and block selection, and the phylogeny was reconstructed using IQTREE with the LG4X matrix and 100 bootstraps. The FASTA file of the alignments and the Newick file used to generate the phylogenetic tree are shown in Supplementary Data Sets 1 and 2.

### Plasmid construction for expression of nonmutant ISA1 and missense mutant variants

Plasmid pBE1343-ISA1 (Facon et al. 2013) is a pET system derivative that expresses the mature ISA1 nonmutant sequence (ISA1-WT) from a codon-optimized synthetic gene driven by the phage T7 promoter. The ISA1 coding sequence of 740 codons begins at the known mature N terminus (Rahman 1998) and is fused at the C terminus to 8 consecutive His codons. pBE1343-ISA1 was modified by PCR-based mutagenesis to introduce the codon changes corresponding to *su1-am*, *su1-Bn2*, *su1-Ref*, *su1-SW*, or *su1-NC* using the primer pairs specified in Supplementary Table S3. Each 50 μL reaction contained 0.2 mm MgSO_4_, 6% DMSO, 0.3 mm each dNTP, 1 μM each primer, 1 ng plasmid template, 1 unit Q5 polymerase (New England Biolabs no. M0491), 1× Q5 polymerase buffer, and 1× Q5 GC enhancer. PCR conditions were 95 °C, 1 min; 55.5 °C, 1 min, 72 °C, 8.5 min for 32 cycle. Following the PCR reaction the products were digested with *Dpn*I for 4 h to remove the template. Product plasmids were purified with the QIAquick PCR Purification Kit (Qiagen no. 28104), then transformed into *E. coli* NEB5-α (New England Biolabs no. C2987H). Each plasmid was sequenced to confirm the mutation and verify integrity of the remainder of the ISA1 open reading frame.

### Expression and partial purification of nonmutant ISA1 and missense mutant variants

pBE1343-ISA1 and the missense mutant variants were each transformed into chemically competent BL21(DE3) expression cells (ThermoScientific no. C600003). Precultures were grown overnight at 37 °C in 10 mL of LB media supplemented with kanamycin at 0.1 mg/mL. A 1 L culture was inoculated with 10 mL of pregrowth culture in LB plus kanamycin, supplemented with 440 mm sorbitol and 2.5 mm betaine. Cultures were grown at 37 °C to OD_600_ of ∼0.8, and then IPTG was added to 0.4 mm to induce expression of the recombinant protein. Induced cultures were grown at 16 °C for ∼16 h. All protein purification steps were performed at 4 °C or on ice. Cell pellets were collected by centrifugation at 5,000 × *g* for 10 min, washed once in H_2_O, resuspended in 100 mL sonication buffer (50 mm Tris-HCl, pH 8.0, 500 mm NaCl, 1 mg/mL lysozyme, 5 mm DTT, 1 mm PMSF, 1× proteinase inhibitor cocktail [Sigma no. P2714], 1% Sarkosyl). Cells were lysed by 15 cycles of sonication for 30 s with 1 min on ice in between each sonication step. The lysate was centrifuged at 15,000 × *g* for 30 min. Supernatants were filtered through 0.45 μm nitrocellulose filters and applied to a 1 mL HisTrapFF affinity column (Cytiva no. 17,531,901) in running buffer (50 mm Tris-HCl, pH 8.0, 500 mm NaCl, 1 mm DTT) at a flow rate of 1 mL/min. The column was washed extensively in running buffer supplemented with 20 mm imidazole. The column was washed further with 60 mm imidazole, and then bound proteins were eluted with 120 mm imidazole.

### α(1→6)-glucosidase assay

Recombinant ISA1 proteins were assayed for α-(1→6)-glucosidase activity at 30 °C in 30 μL of 50 mm sodium phosphate buffer, pH 6.0, containing 5 mg/mL oyster glycogen (Sigma no. G8751). Samples of 10 μL were removed from the assay at 5 min intervals over 30 min total, and added to 10 μL of 0.1 m NaOH in a 96 well microtiter plate to stop the reaction. Reducing ends were quantified by the copper-bicinchoninate method (Fox and Robyt 1991). Each well received 80 μL of H_2_O and 150 μL of a 1:1 mixture of solutions A and B described in the referenced method. The plates were covered with plastic wrap and incubated for 30 min at 80 °C. After cooling to room temperature, the absorbance at 560 nm was recorded for each well.

Enzyme concentration was adjusted so that the increase in A_560_ was linear over the 20 to 40 min period of the assay. This quantity amounted to ∼10 μg. In most instances enzymes were assayed on the same day that they were purified from induced *E. coli* cultures. Blank assays with no enzyme provided negative controls. Positive controls were provided by approximately 0.25 U of commercial ISA from *Pseudomonas* (Megazyme no. E-ISAMY), assayed similarly with the exception that the buffer was 50 mm sodium acetate, pH 4.8. Standard curves were obtained with maltose ranging from zero to 2.1 μg to ensure linearity between A_560_ and reducing end concentration. Standard curves, and negative and positive controls, were run each day in the same microtiter plate as the ISA assay.

### Structural modeling

Modeling of ISA1-ISA2 heterotetramer was performed using AlphaFold3 (Abramson et al. 2024). To predict glucan-binding sites on maize ISA1-ISA2 heterotetramer, each promoter of the ISA1-ISA2 model was structurally aligned with the *Chlamydomonas* ISA1 crystal structure bound to maltoheptaose (PDB 4OKD). Following alignment, the *Chlamydomonas* ISA1 model was then removed leaving only the bound glucans. For ISA2 models, bound glucans were modeled only at SBS1 as described in the *Results* section.

For visualization, the glucan binding sites were also represented as extended cylindrical tubes approximating the diameter of the glucan chain. This was achieved by aligning 2 or 4 molecules of the maltoheptaose from the *Chlamydomonas* ISA1 structure (PDB 4OKD) generating a 14- or 28-glucose unit of α-polyglucan models. Simulated density maps were generated from these extended glucan models using the “Fit in Map” tool in UCSF Chimera. The resulting tubes were then manually positioned within the predicted glucan-binding grooves of the ISA1-ISA2 complex to overlay with the original maltoheptaose ligands. A 14-glucose unit glucan was used to represent binding at SBS1 and SBS2, while a 28-glucose unit model was used to represent binding at the central channel.

### Analyses of ISA1 protein levels and ISA activity in endosperm extracts

Preparation of soluble endosperm extracts was performed as previously described (Kubo et al. 2010) with the following modifications. Extraction buffer was 50 mm Tris-acetate, pH 7.5, 5 mm MgCl_2_, 1 mm DTT, 1 mm PMSF, 0.15% (v/v) Tween-20, 1× Protease Inhibitor Cocktail (Sigma no. P2714). Clarification by centrifugation was for 15 min at full speed in a microfuge. Supernatants were passed through a 0.45 μM nitrocellulose syringe tip filter. Total protein concentration in the extracts was determined by Bradford assay. For immunoblot analysis, 30 μg total soluble protein was fractionated by SDS-PAGE in 1.5 mm thick gels in a Bio-Rad Mini-Protean cell. Gels were electroblotted to nitrocellulose membranes (Thermo iBlot2), and transferred proteins were probed with purified IgG that binds specifically to ISA1, referred to as α-ISA1. This antibody was raised against a peptide sequence predicted to be unique to ISA1 (Kubo et al. 2010). The peptide antigen was used as an affinity ligand to purify IgG specific for that ISA1 epitope from crude rabbit serum, as previously described (Hennen-Bierwagen et al. 2008). Specificity α-ISA1 was demonstrated by lack of any signal of the correct molecular mass in the *su1-4582* null mutant that fails to express any ISA1 protein (Fig. 6A). α-ISA1 was diluted 1/1,000 for use as the immunoblot probe. IgG binding was detected by goat anti-rabbit secondary antibody conjugated to horseradish peroxidase (Thermo no. 31,460), at 1/15,000 dilution. Bound secondary antibody was visualized by chemiluminescence (Thermo no. 34,580).

In-gel enzyme activity assays were performed as previously described (Dinges et al. 2001; Kubo et al. 2010). Total soluble extract (25 or 50 μg) was fractionated by native PAGE, then those gels were electroblotted to a second native gel cast in buffer conditions conducive to enzyme activity and containing 3% solubilized potato starch embedded in the acrylamide matrix. This electroblotting step utilized a standard system in which the 2 gels are fully immersed in transfer buffer in a tank with transversely mounted electrodes. After transfer the gels are stained with iodine solution to reveal the purple background of the embedded starch and colored bands where the structure of that material had been altered by an enzyme such that the emission spectrum of the iodine complex had changed. Identification of the proteins responsible for each colored band was previously described (Colleoni et al. 2003; Kubo et al. 2010).

### Size exclusion chromatography

All steps were conducted on ice or at 4 °C except as noted. Ten to twelve endosperms dissected from kernels harvested 20 DAP and stored at −80 °C were ground in mortar and pestle in 2 mL of extraction buffer (50 mm Tris-acetate, pH 7.5, 5 mm MgCl_2_, 1 mm DTT, 1 mm PMSF, 0.15% Tween-20, 1× proteinase inhibitor cocktail [Sigma no P2714]). Starch was removed by centrifugation at 2,400 × *g* for 10 min in a microfuge. The supernatant was centrifuged again, at 17,000 × *g* in a microfuge for 10 min. Prior to this second centrifugation the *su1-Ref* extract was treated with 10 μL of heat stable α-amylase from *Bacillus licheniformis* (Sigma no. A3306) for 20 min at room temperature to degrade phytoglycogen. After centrifugation the supernatants were filtered through a 0.45 μm nitrocellulose filter, then 0.5 mL containing 2 to 3 μg of total protein were applied to a Superose 6 Increase 10/300 GL size exclusion column. The column was eluted in 50 mm Tris-acetate, pH 7.5, 150 mm NaCl, 1 mm DTT at a flow rate of 0.5 mL/min while 0.5 mL fractions were collected. Samples of selected fractions (30 μL) were separated by SDS-PAGE and probed in immunoblot analysis with αISA1-IgG.

### Statistical analyses

For correlation analysis (Fig. 8B), sucrose and phytoglycogen content data were log_10_-transformed to address heteroscedasticity and non-normality. The resultant transformed dataset was used to calculate Spearman's rank correlation between sucrose and phytoglycogen levels using the cor.test() function in R (R Core Team 2024). Pairwise comparison of data sets to determine *P* values used the Student's *t*-test function in Microsoft Excel with options for 2 tailed distributions and 2-sample comparisons with equal variance (Supplementary Data Sets 3 and 4).

### Accession numbers

Sequence data from this article can be found in the GenBank/EMBL data libraries under accession numbers shown in the legend to Figs. 2 and 11, Supplementary Figs. S2, S10 and S11, and in the gene model in the maize B73 genome sequence noted in Table 1.

## Supplementary Material

koaf220_Supplementary_Data

## Data Availability

The data underlying this article are available in the article and in its online supplementary material.

## Supplementary data

The following materials are available in the online version of this article.

**Supplementary Figure S1.** Expanded phylogenetic analysis of GH13_11 proteins.

**Supplementary Figure S2.** Structures of α-amylase superfamily members.

**Supplementary Figure S3.** Maize pedigrees.

**Supplementary Figure S4.** Haplotypes of the maize *su1* locus and positions of mutant alleles.

**Supplementary Figure S5.** Biological replicates of the amylopectin chain length distribution analysis from *su1-Ref* lines.

**Supplementary Figure S6.** Phytoglycogen chain length distribution analysis from *su1-Ref* lines.

**Supplementary Figure S7.** Amylopectin chain length distribution analysis from *su1-Bn2* lines or *su1-st* lines.

**Supplementary Figure S8.** Size exclusion chromatography.

**Supplementary Figure S9.** Closeup structural model of ISA1 showing the location of missense substitution sites.

**Supplementary Figure S10.** Sequence and structural conservation of SBS1 in ISA2.

**Supplementary Figure S11.** Comparison of CBM48 structure between maize ISA1 and Arabidopsis SEX4.

**Supplementary Table S1.** Seed stocks available for distribution.

**Supplementary Table S2.** PCR primers for amplification and sequencing of the *su1* genomic locus.

**Supplementary Table S3.** PCR primers for mutagenesis of the ISA1 coding sequence.

**Supplementary Data Set 1.** FASTA file of alignments used to generate the phylogeny shown in Fig. 1 and Supplementary Fig. S1.

**Supplementary Data Set 2.** Newick file use the generate the phylogeny shown in Fig. 1 and Supplementary Fig. S1.

**Supplementary Data Set 3.** Statistical treatment for polyglucan content (Supports Fig. 5).

**Supplementary Data Set 4.** Statistical treatment for sucrose content (Supports Fig. 8).

## Dive Curated Terms

The following phenotypic, genotypic, and functional terms are of significance to the work described in this paper:

ISA3 Gramene: AT4G09020
ISA3 Araport: AT4G09020

SEX4 Gramene: AT3G52180
SEX4 Araport: AT3G52180

ISA Gramene: ostta02g05060
ISA Araport: ostta02g05060

starch CHEBI: CHEBI:28017

SBE Gramene: ostta03g00870
SBE Araport: ostta03g00870

amylopectin CHEBI: CHEBI:28057

PTST2 Gramene: AT1G27070
PTST2 Araport: AT1G27070
