# Supplementary material for: Noncatalytic functions of ISOAMYLASE 1 and 2 affect the proportion of insoluble and soluble α-polyglucans in maize
Source: Plant Cell. 2025 Sep 22;37(10):koaf220. doi: 10.1093/plcell/koaf220 (PMC12510319; doi:10.1093/plcell/koaf220)
Supplement: koaf220_Supplementary_Data [file koaf220_supplementary_data.zip › Supp Combined.pdf]

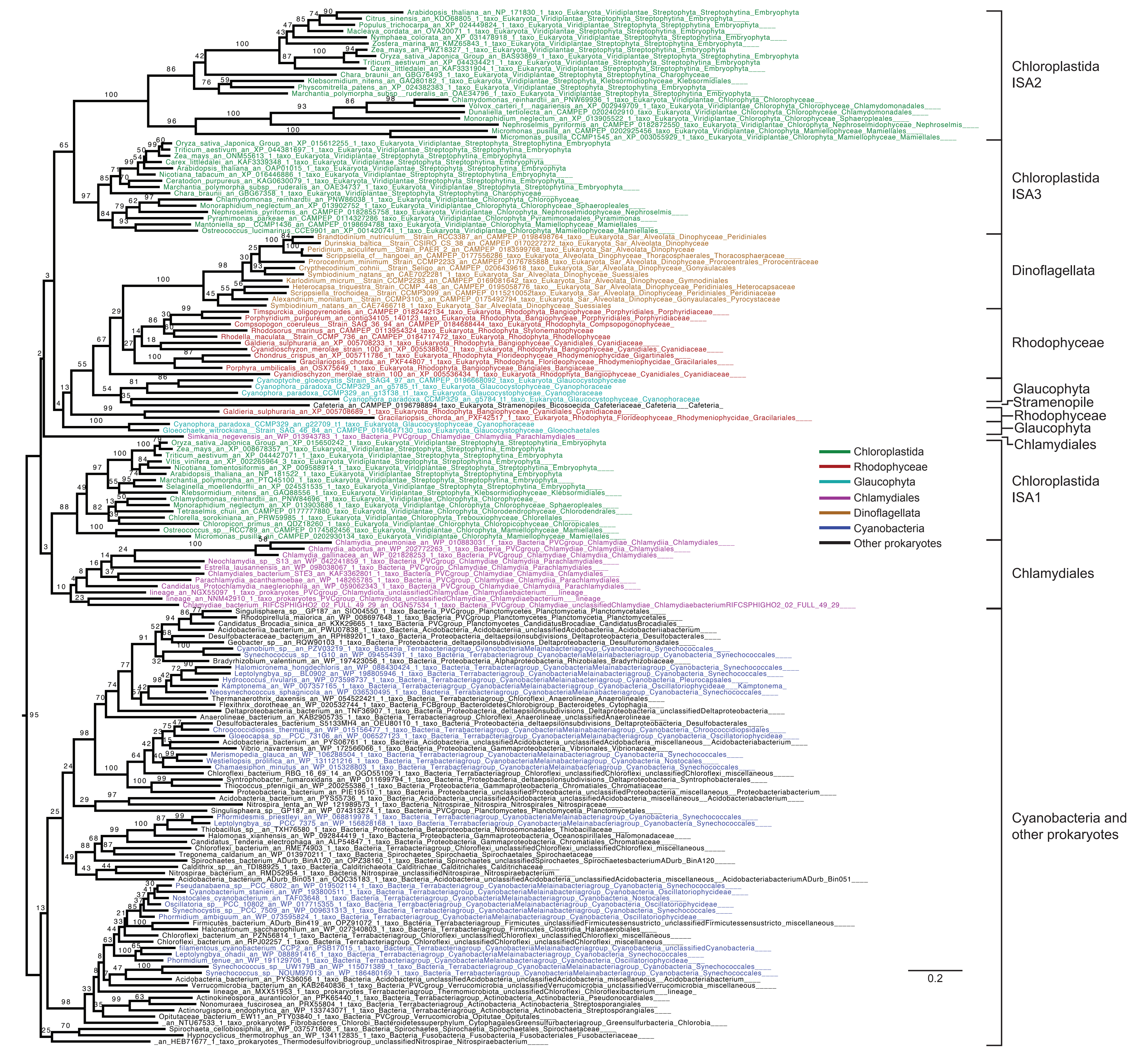

Supplementary Figure S1. Rooted midpoint phylogenetic tree (Supports Figure 1). Species and database identifiers for each sequence are indicated and all bootstrap values are shown. Phylogenetic classification indicated by colored text and labels corresponds to Figure 1. The scale bar shows the inferred number of amino acid substitutions per site.

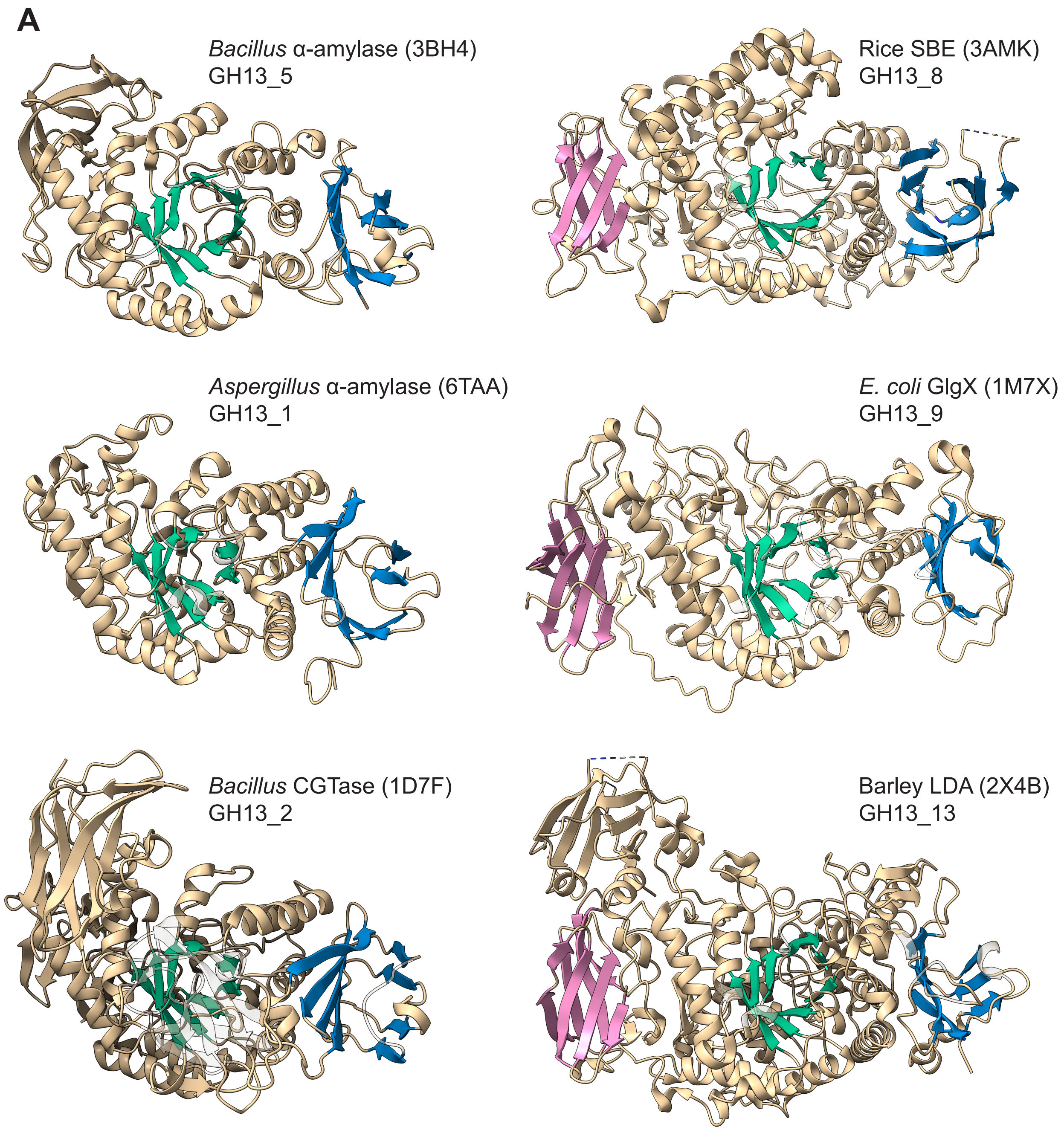

Supplementary Figure S2. Structures of α-amylase superfamily members (Supports Figure 3). Crystal structures are indicated by Protein Data Bank (PDB) identifiers in parentheses. Structures without PDB identifiers are AlphaFold3 predictions. GH13 subfamilies are indicated. **A)** GH13 family enzymes other than GH13_11. The catalytic domain colored bluish green and the β-sandwich domain colored blue are present in all members of superfamily. The CBM48 domain colored reddish purple is present in GH13 enzymes that create or hydrolyze α-(1→6) glycoside bonds. **B)** GH13_11 proteins. Domain colors correspond to panel A. The β sandwich domain colored orange is specific to ISA2. Genbank accession numbers for primary sequences analyzed by AlphaFold3 are: Maize ISA1, ACG43008; Maize ISA2, PWZ18327; *Chara braunii* ISA2, GBG76493; *Chlamydomonas reinhardtii* ISA2, PNW69936; *Ceratodon purpureus* ISA2, KAG0562914.

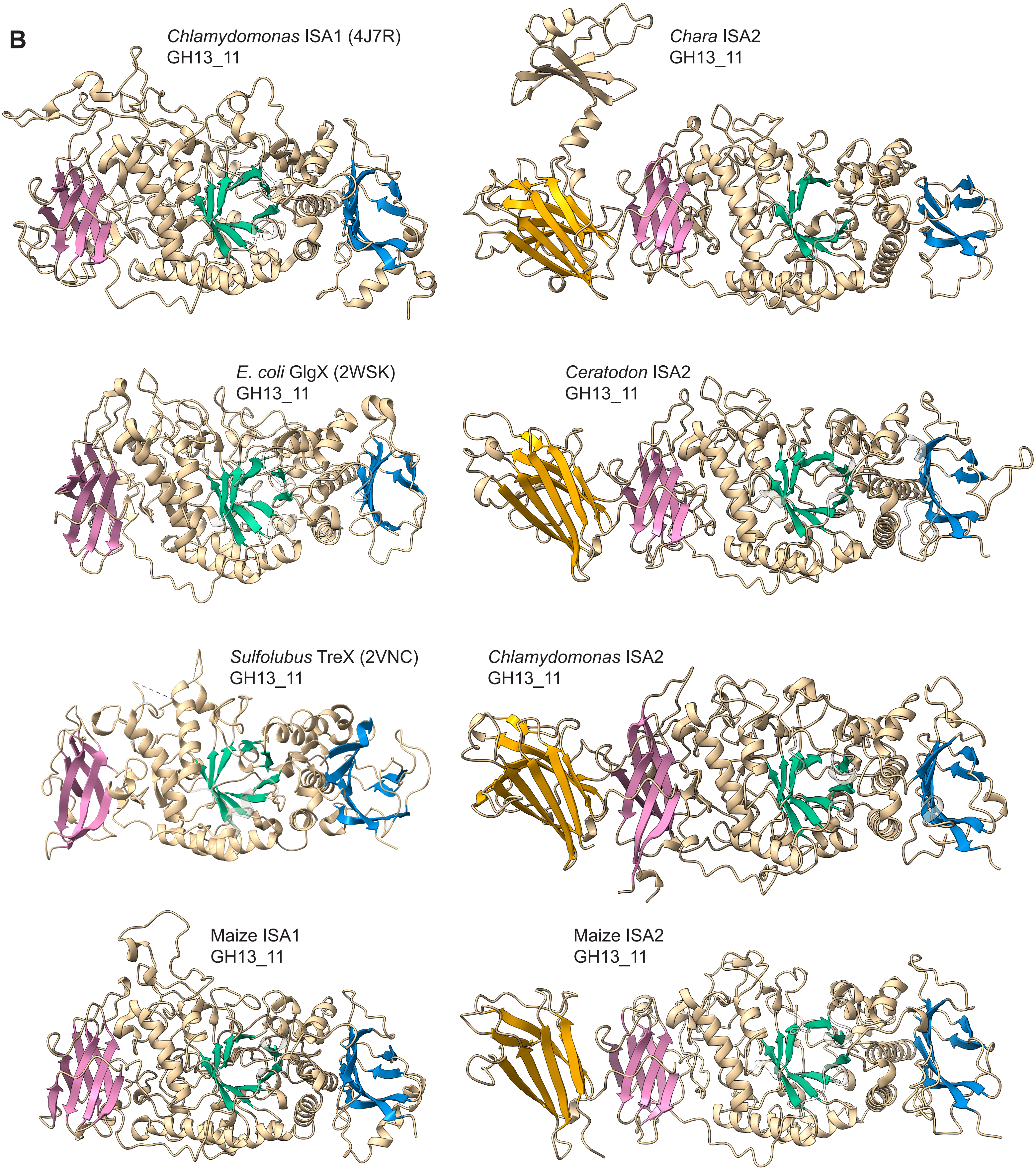

B
Chlamydomonas ISA1 (4J7R)
GH13_11
Chara ISA2
GH13_11
E. coli GlgX (2WSK)
GH13_11
Ceratodon ISA2
GH13_11
Sulfolubus TreX (2VNC)
GH13_11
Chlamydomonas ISA2
GH13_11
Maize ISA1
GH13_11
Maize ISA2
GH13_11

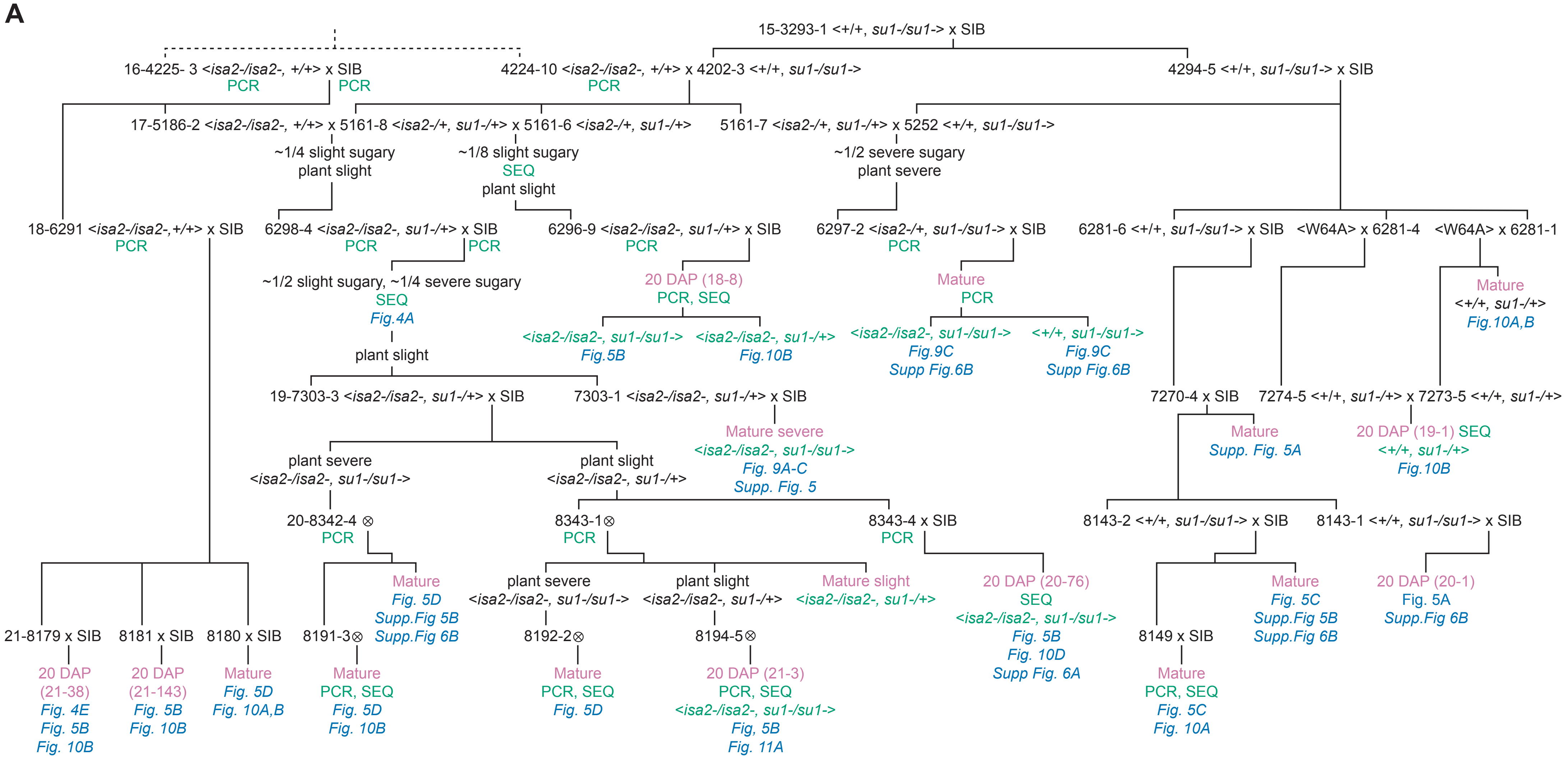

Supplementary Figure S3, Page 1

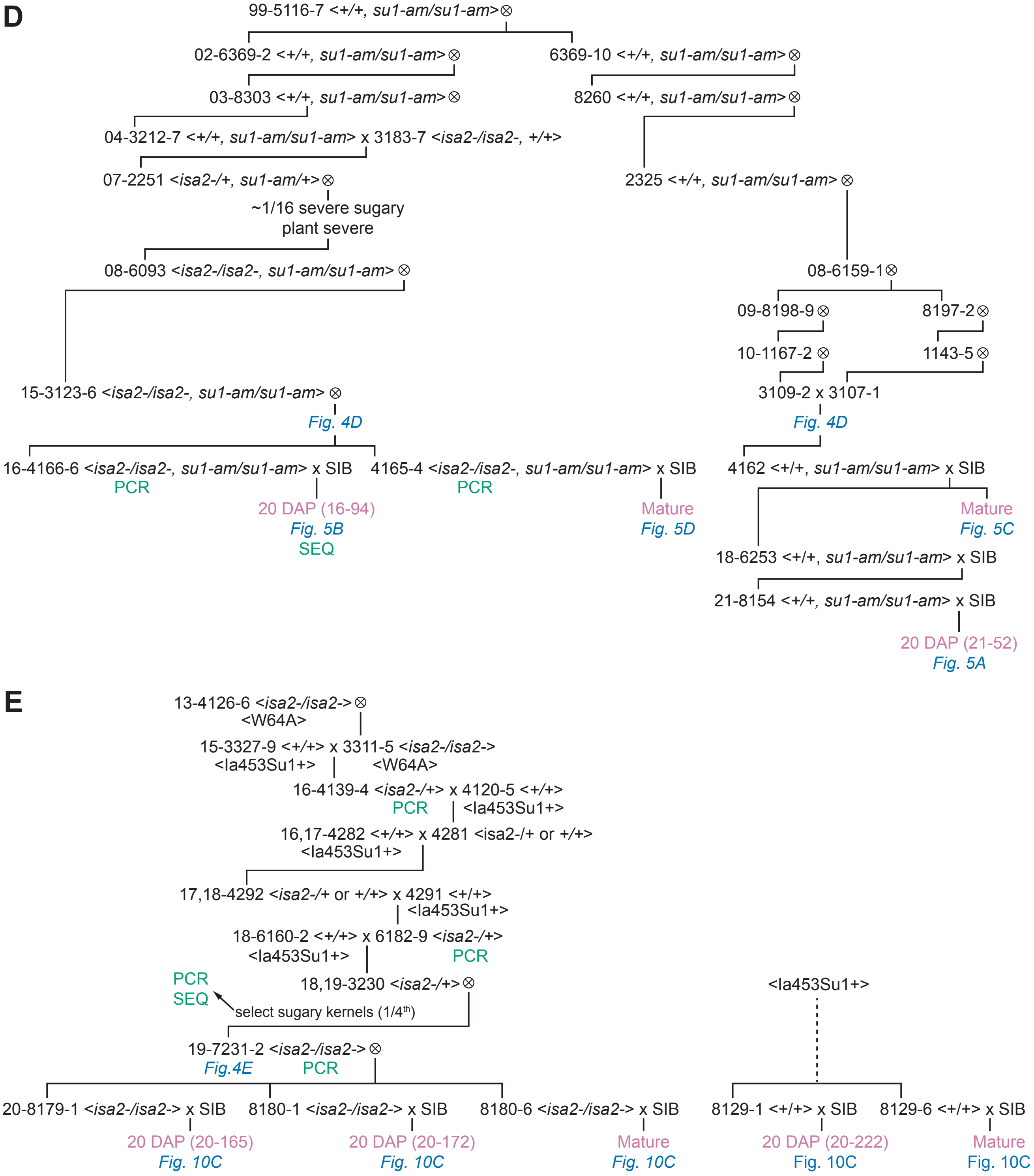

Supplementary Figure S3. Maize pedigrees (Supports Figures. 4 -10). Plants from each nursery year are aligned horizontally. Genoytpes were confirmed in the indicated plants or kernels by PCR analysis for the *isa2* locus (PCR - bluish green) and by genomic DNA sequencing for the *su1* locus (SEQ - bluish green). Redish purple text indicates kernel samples and the developmental stage of harvest, with numbers in parentheses indicating frozen stock identifiers. Blue text shows figures from the manuscript or supplemental data that include the indicated samples. **A)** Lineages including *isa2-339* and *su1-Ref*. Dotted lines indicate a common ancestor removed by more than one generation. **B)** Lineages including *isa2-339* and *su1-st*. **C)** Lineages including *isa2-339* and *su1-Bn2*. **D)** Lineages including *isa2-339* and *su1-am*. **E)** Introgression of *isa2-339* into the otherwise non-mutant Ia453Su1+ genetic background.

B

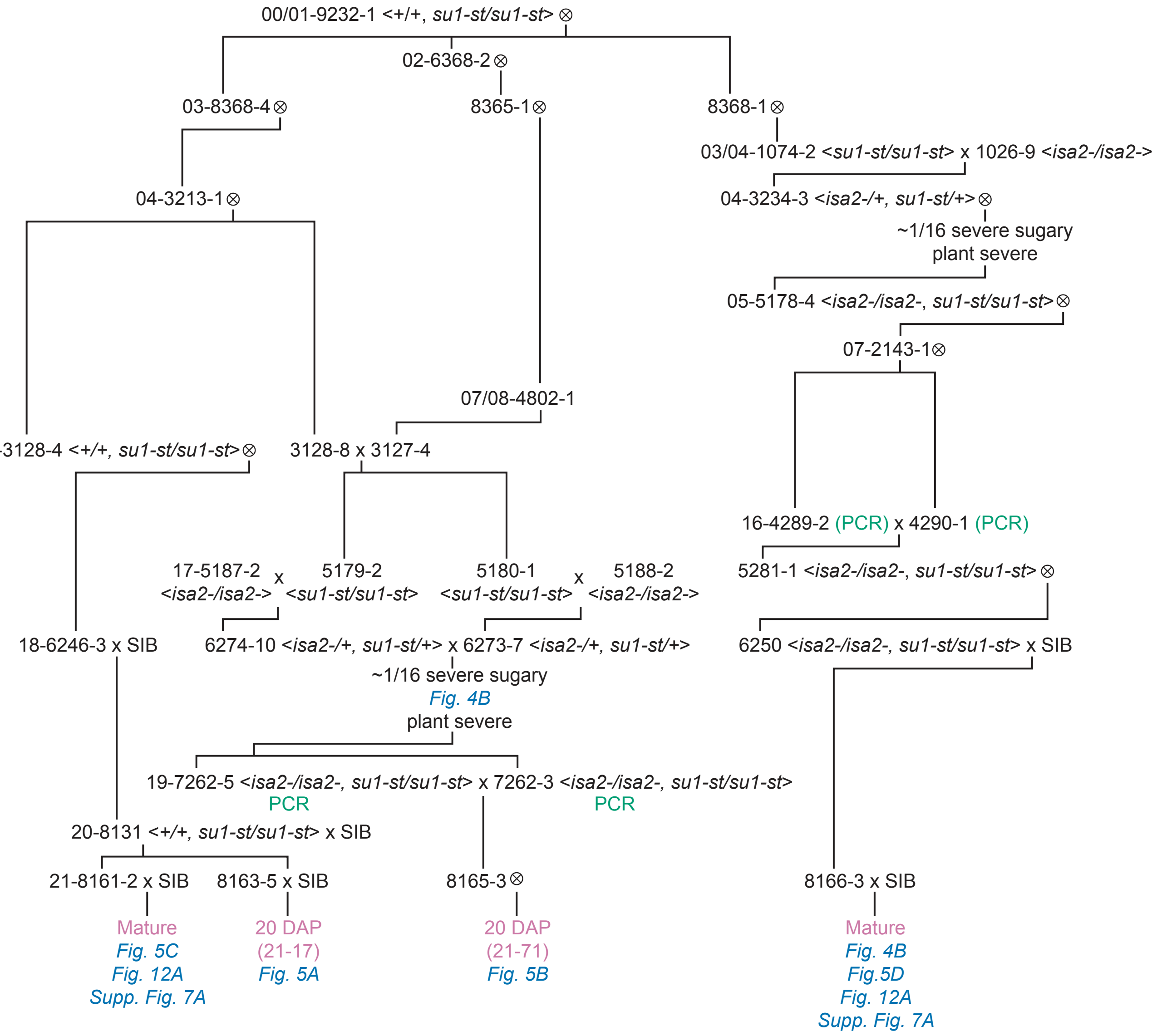
00/01-9232-1 <+/+, *su1-st/su1-st*> ⊗
02-6368-2⊗
03-8368-4⊗
8365-1⊗
8368-1⊗
03/04-1074-2 <*su1-st/su1-st*> x 1026-9 <*isa2-/isa2-*>
04-3213-1⊗
04-3234-3 <*isa2-/+, su1-st/+*>⊗
~1/16 severe sugary
plant severe
05-5178-4 <*isa2-/isa2-, su1-st/su1-st*>⊗
07-2143-1⊗
07/08-4802-1
15-3128-4 <+/+, *su1-st/su1-st*>⊗
3128-8 x 3127-4
16-4289-2 (PCR) x 4290-1 (PCR)
17-5187-2
<*isa2-/isa2-*>
x
5179-2
<*su1-st/su1-st*>
5180-1
<*su1-st/su1-st*>
x
5188-2
<*isa2-/isa2-*>
5281-1 <*isa2-/isa2-, su1-st/su1-st*>⊗
18-6246-3 x SIB
6274-10 <*isa2-/+, su1-st/+*> x 6273-7 <*isa2-/+, su1-st/+*>
6250 <*isa2-/isa2-, su1-st/su1-st*> x SIB
~1/16 severe sugary
Fig. 4B
plant severe
19-7262-5 <*isa2-/isa2-, su1-st/su1-st*> x 7262-3 <*isa2-/isa2-, su1-st/su1-st*>
PCR
PCR
20-8131 <+/+, *su1-st/su1-st*> x SIB
21-8161-2 x SIB
8163-5 x SIB
8165-3⊗
8166-3 x SIB
Mature
Fig. 5C
Fig. 12A
Supp. Fig. 7A
20 DAP
(21-17)
Fig. 5A
20 DAP
(21-71)
Fig. 5B
Mature
Fig. 4B
Fig.5D
Fig. 12A
Supp. Fig. 7A

C

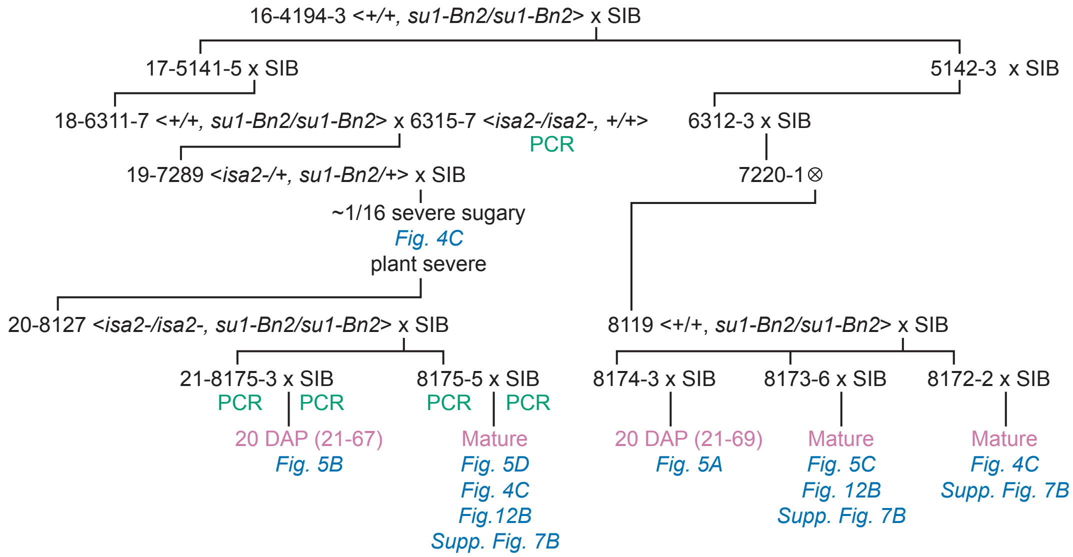
16-4194-3 <+/+, *su1-Bn2/su1-Bn2*> x SIB
17-5141-5 x SIB
5142-3 x SIB
18-6311-7 <+/+, *su1-Bn2/su1-Bn2*> x 6315-7 <*isa2-/isa2-, +/+*>
PCR
6312-3 x SIB
19-7289 <*isa2-/+, su1-Bn2/+*> x SIB
7220-1⊗
~1/16 severe sugary
Fig. 4C
plant severe
20-8127 <*isa2-/isa2-, su1-Bn2/su1-Bn2*> x SIB
8119 <+/+, *su1-Bn2/su1-Bn2*> x SIB
21-8175-3 x SIB
PCR
PCR
8175-5 x SIB
PCR
PCR
8174-3 x SIB
8173-6 x SIB
8172-2 x SIB
20 DAP (21-67)
Fig. 5B
Mature
Fig. 5D
Fig. 4C
Fig.12B
Supp. Fig. 7B
20 DAP (21-69)
Fig. 5A
Mature
Fig. 5C
Fig. 12B
Supp. Fig. 7B
Mature
Fig. 4C
Supp. Fig. 7B

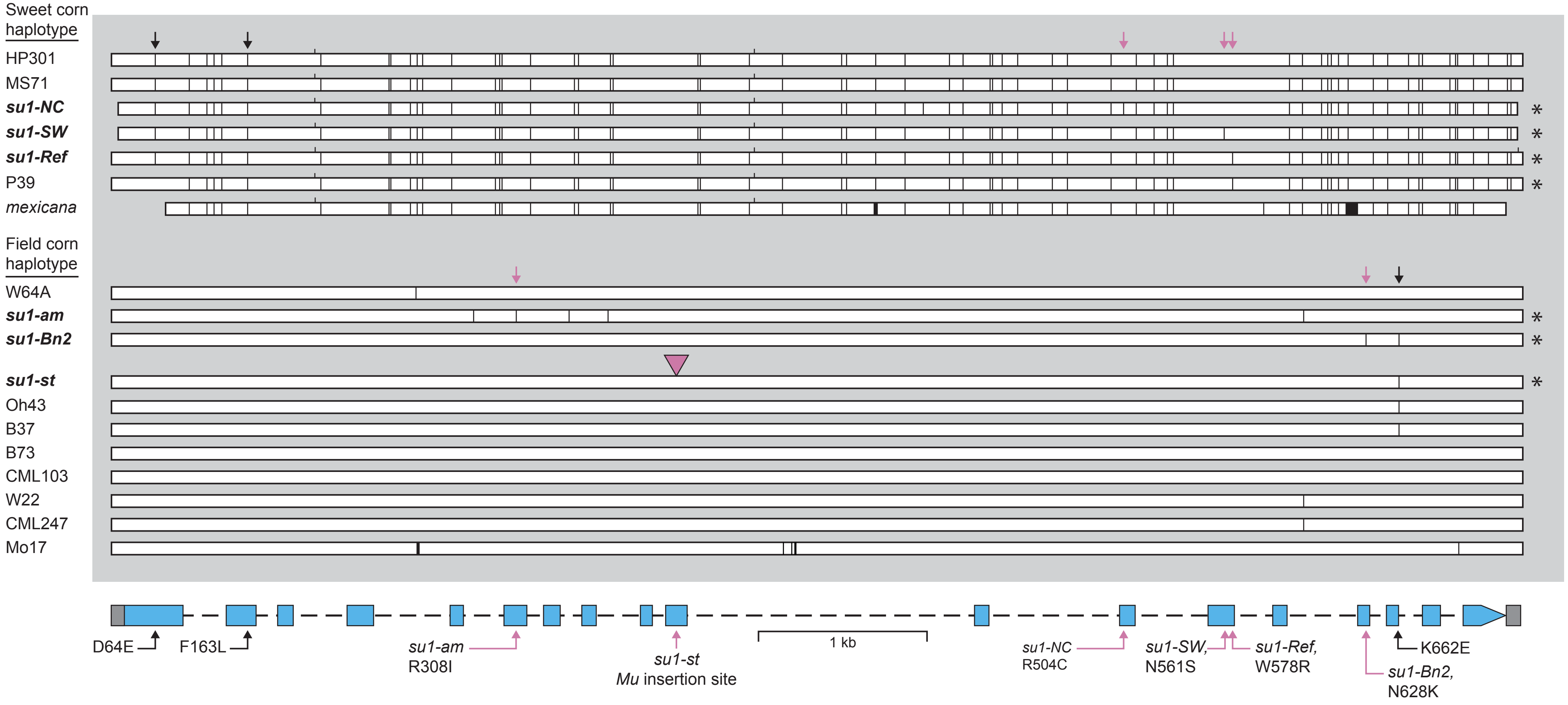

Supplementary Figure S4. Haplotypes of the *su1* locus and positions of mutant alleles (Supports Table 1). The genomic sequences of *su1-Bn2*, *su1-st*, and *su1-am* were compared to previously described sequences of the *su1* locus from various non-mutant inbreds or mutant lines (Hu *et al.*, 2021). White space indicates identity with the reference non-mutant allele from inbred B73 and vertical lines indicates single nucleotide polymorphishsms or insertion/deletions of less than 5 bp. The reddish purple triangle indicates insertion of a *Mutator* transposon in the *su1-st* allele. Asterisks indicate *su1-* mutant alleles. Arrows represent amino acid differences relative to B73, with black color indicating variants that occur in non-mutant lines and reddish purple color indicating mutant alleles. The position of each amino acid variation in the ISA1 coding sequence is indicated in the gene model, in which blue boxes indicate coding sequence, grey boxes indicate untranslated regions, and dotted lines represent introns. The figure is drawn to scale.

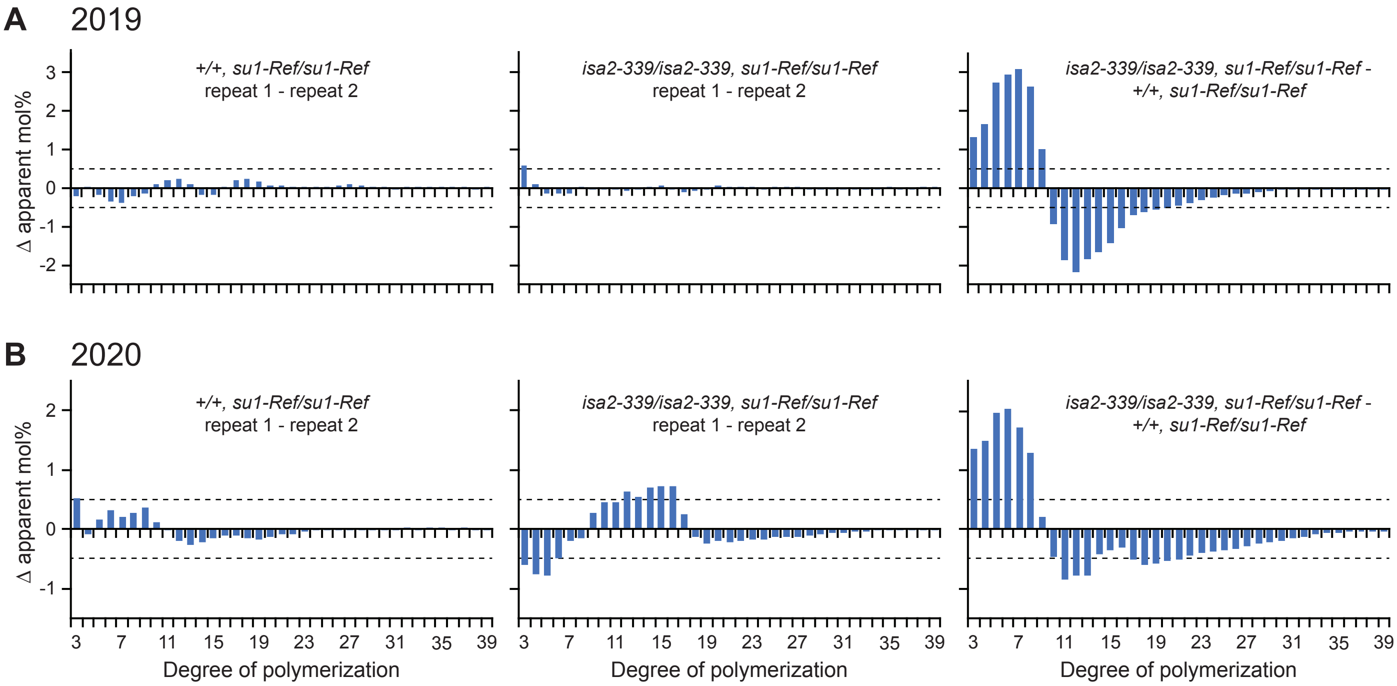

Supplementary Figure S5. Biological replicates of the amylopectin chain length distribution analysis from *su1-Ref* lines (Supports Figure 9). All kernels are in the W64A inbred background. Biological repeats are analyses of separate isogenic single kernels. The average of the two biological repeats was used to compare between genotypes. Dotted lines indicate 0.5% difference, assigned as the limit of technical variation. **A)** Kernels from separate homozygous ears, 2019 field season. **B)** Kernels from separate homozygous ears, 2020 field season.

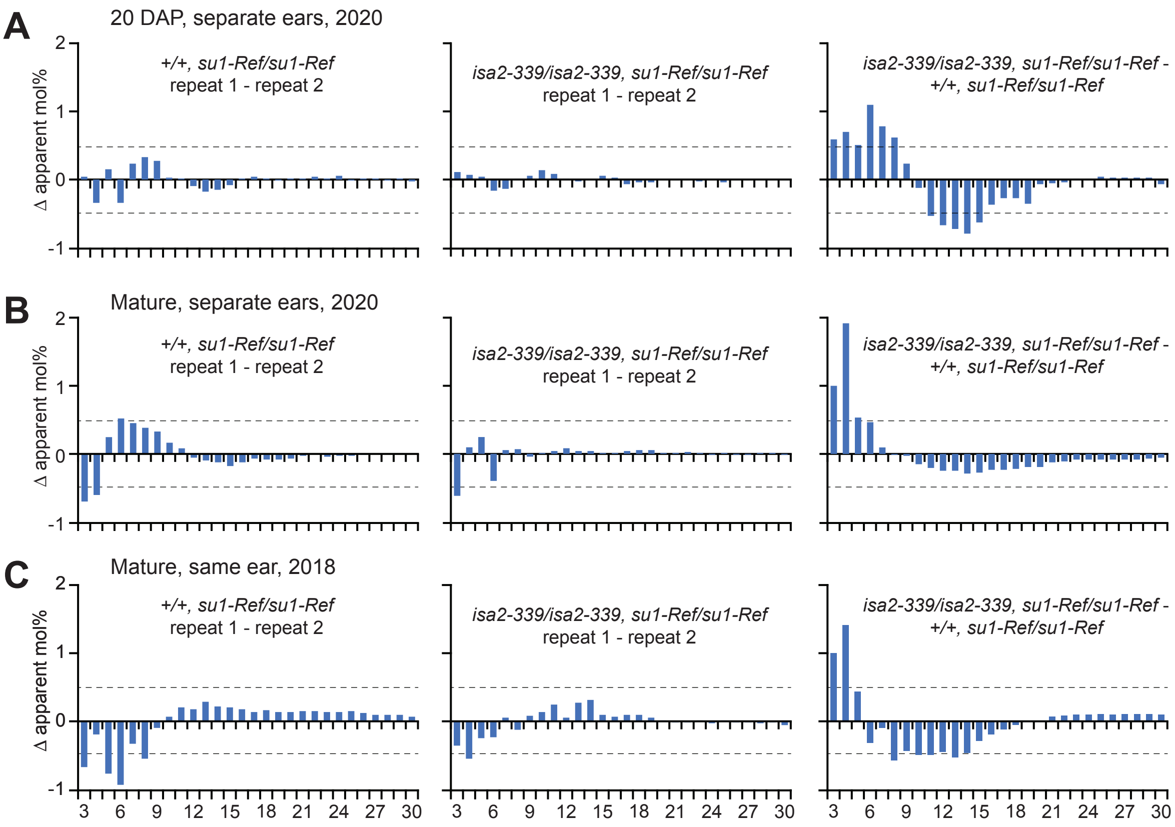

Supplementary Figure S6. Phytoglycogen linear chain length distribution differences from *su1-Ref* lines (Supports Figure 9). All kernels are in the W64A inbred background. Biological repeats are analyses of separate isogenic single kernels. The average of the two biological repeats was used to compare between genotypes. Dotted lines indicate 0.5% difference, assigned as the limit of technical variation. All plots are at the same scale. **A)** Immature kernels harvested 20 DAP from separate ears, 2020 field season. **B)** Mature kernels from separate homozygous ears, 2020 field season. **C)** Mature kernels from a segregating ear, 2018 field season.

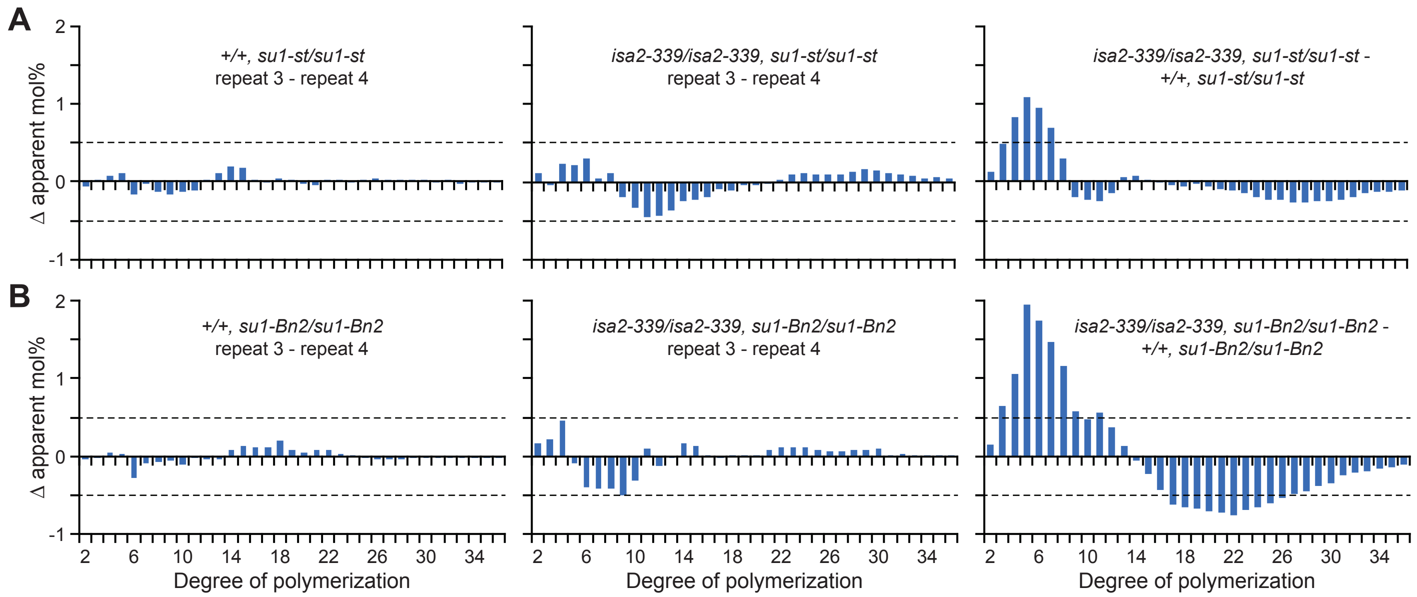

Supplementary Figure S7. Amylopectin chain length distribution analysis from *su1-Bn2* lines or *su1-st* lines (Supports Figure 9). All kernels are in the W64A inbred background and are homozygous for the indicated mutation(s). Biological repeats are analyses of separate isogenic single kernels. The average of the two biological repeats was used to compare between genotypes. Dotted lines indicate 0.5% difference, assigned as the limit of technical variation. All plots are at the same scale. **A)** Effect of *isa2-339* in the *su1-st* genetic background. **B)** Effect of *isa2-339* in the *su1-Bn2* genetic background.

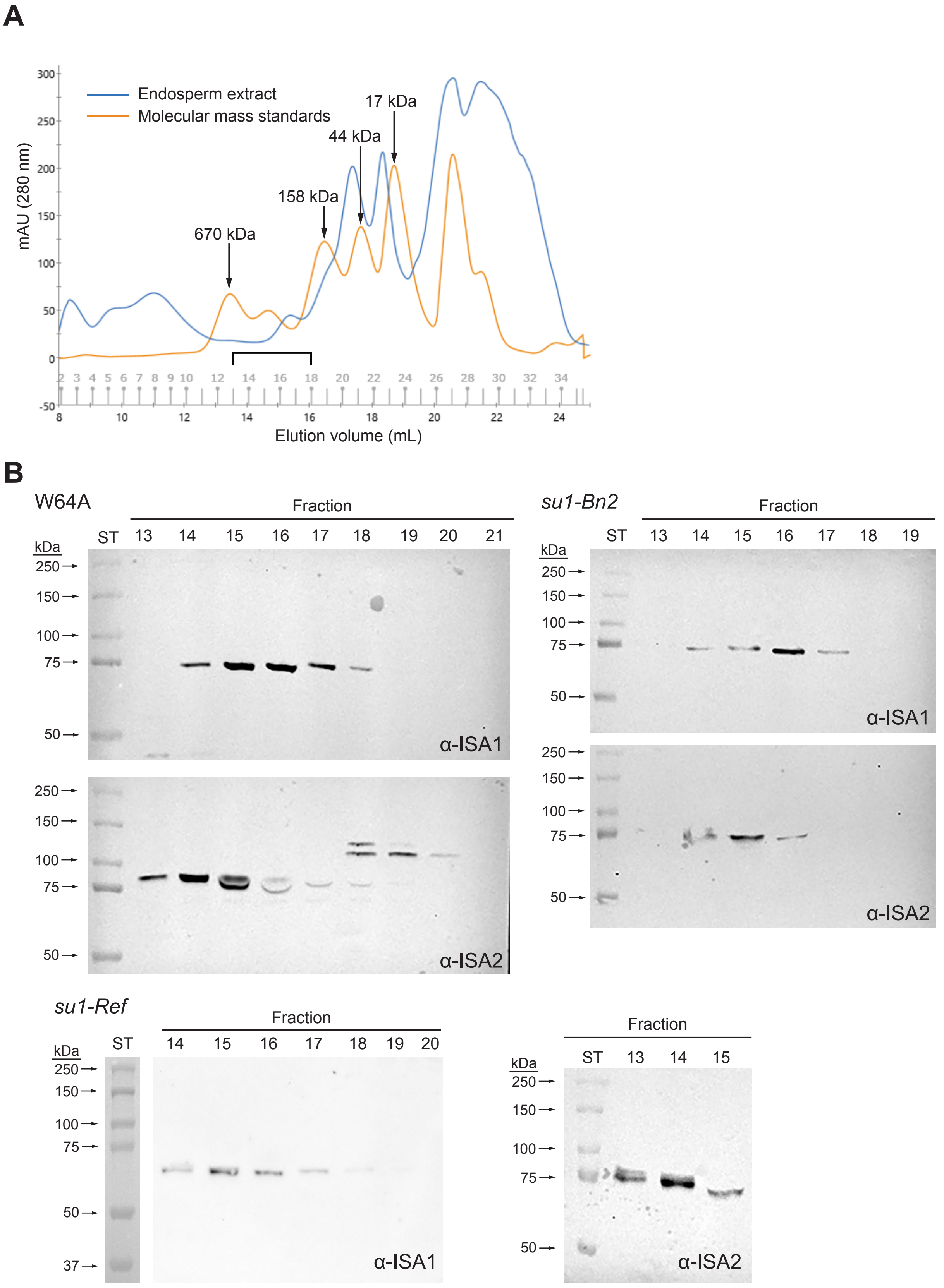

Supplementary Figure S8. Size exclusion chromatography (SEC) (Supports Figure 11). **A)** SEC elution profile for *su1-Ref* endosperm extract and molecular mass standards subjected to the identical chromatography protocol. Essentially identical results were obtained for all genotypes. The black bracket indicates fractions containing ISA1 and ISA2 as identified in panel B. **B)** Immunoblot analysis of SEC fractions. Endosperm extracts from tissue harvested 20 DAP were fractionated by SEC. Selected fractions were further fractionated by SDS-PAGE and probed with α-ISA1 IgG or α-ISA2 IgG, as indicated. Fraction numbers correspond to panel A. Genotypes are indicated. "ST" indicates molecular weight standards.

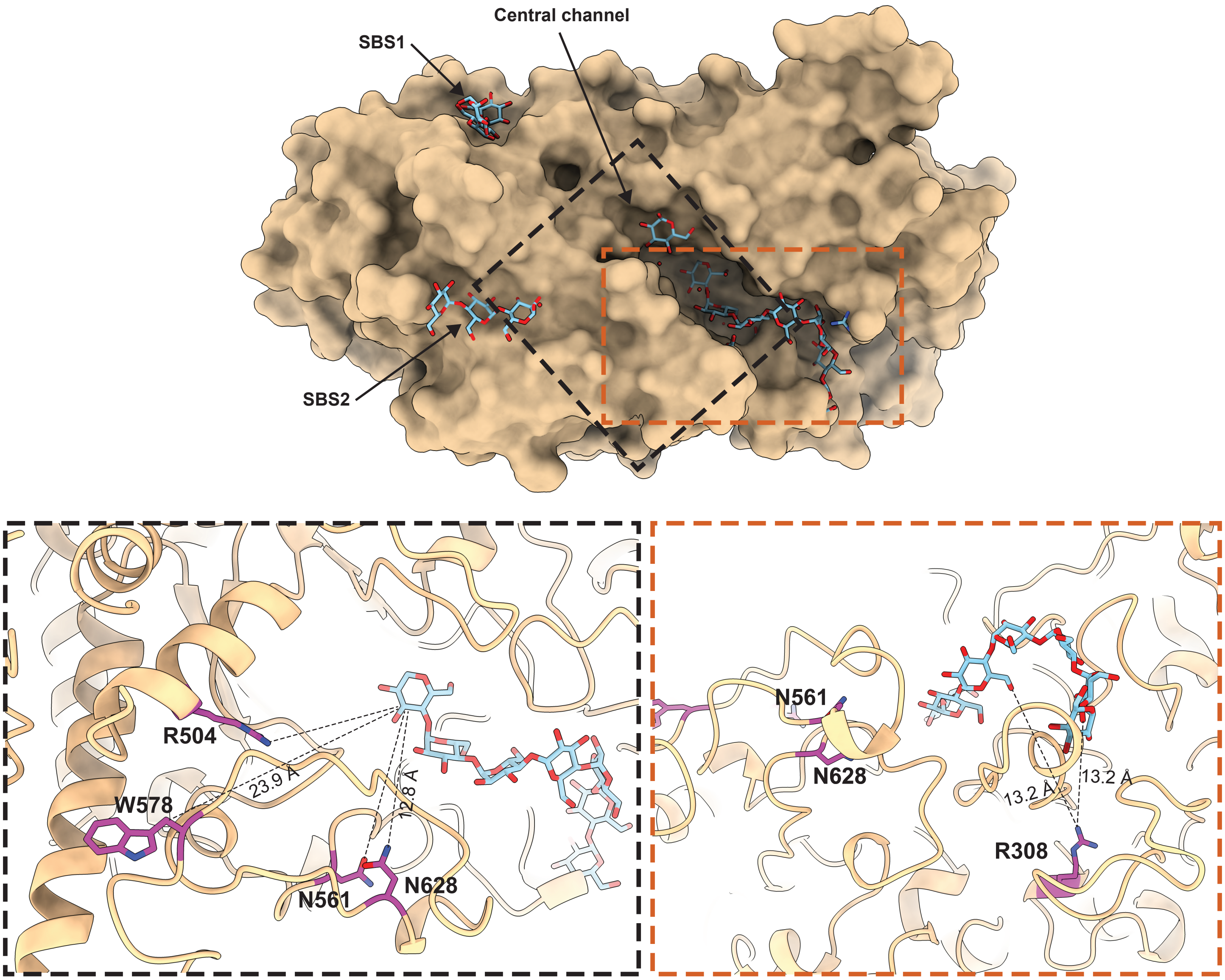

Supplementary Figure S9. Closeup structural model of ISA1 showing the location of missense substitution sites (Supports Figure 11). Magnified regions correspond to the areas indicated by dotted lines of the same color in the surface model. Positioning of maltoheptaose bound to *Chlamydomonas* ISA1 (PDB identifier 4OKD) in the maize homolog was performed as in Fig. 11A. Correspondence between alleles and mutated residues (purple color) is shown in Table 1.

A

ISA2, SBS1

```
                         757          767
Chlamydomonas ISA1     DIQWHGE-LPNTPDWTD
Chlamydomonas ISA2     EVAWHSPYGGGEPDWSG
Arabidopsis ISA2       NIVWYAN-DQTTPKWED
Poplar ISA2            NIDWHGS-DQNPPRWED
Ceratopteris ISA2      NIQWHGL-EINQPDWEN
Selaginella ISA2       MITWHGATYEEEPLWTD
Klebsormidium ISA2     ELVWHGY-WPAHPEWDN
Physcomitrium ISA2     RLTWHGL-KPEQPLWEC
Chlorella ISA2         DIRWHSVDAAAAPVWEA
Maize ISA2             NIHWYGS-DLSEPCWED
```

B

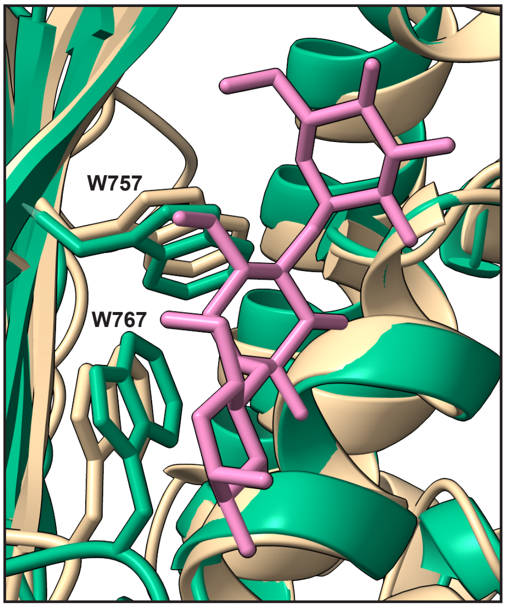

Supplementary Figure S10. Sequence and structural conservation of SBS1 in ISA2 (Supports Figures 11 and 12). **A)** Primary sequence conservation surrounding the glucan contact sites in SBS1. ISA2 sequences from the indicated species are compared to SBS1 of Chlamydomonas ISA1. Blue color indicates glucan contact residues. Bold black text indicates a residue conserved in all proteins included in the analysis. Residue numbers are indicated for *Chlamydomonas* ISA1. GenBank identifiers of the squences analyzed are: Chlamydomonas ISA1, AAP85534; Chlamydomonase ISA2, PNW69936; Arabidopsis ISA2, NP_171830; Poplar ISA2, XP_024449824; Ceratopteris ISA2, KAH7435504; Selaginella ISA2, XP_024535142; Klebsormidium ISA2, GAQ80182; Physcomitrium ISA2, XP_024382383; Chlorella ISA2, KAI3428209; Maize ISA2, PWZ18327. **B)** Structural conservation of SBS1. Tan color indicates the *Chlamydomonas* ISA1 crystal structure (PDB identifier 4OKD) and bluish green color indicates the maize ISA2 structure predicted by AlphaFold3. Reddish purple color indicates glucosyl residues in maltoheptaose modeled from the Chlamydomonas ISA1 crystal structure. Residues that directly contact bound carbohydrate are numbered according to the Chlamydomonas ISA1 primary sequence shown in panel A.

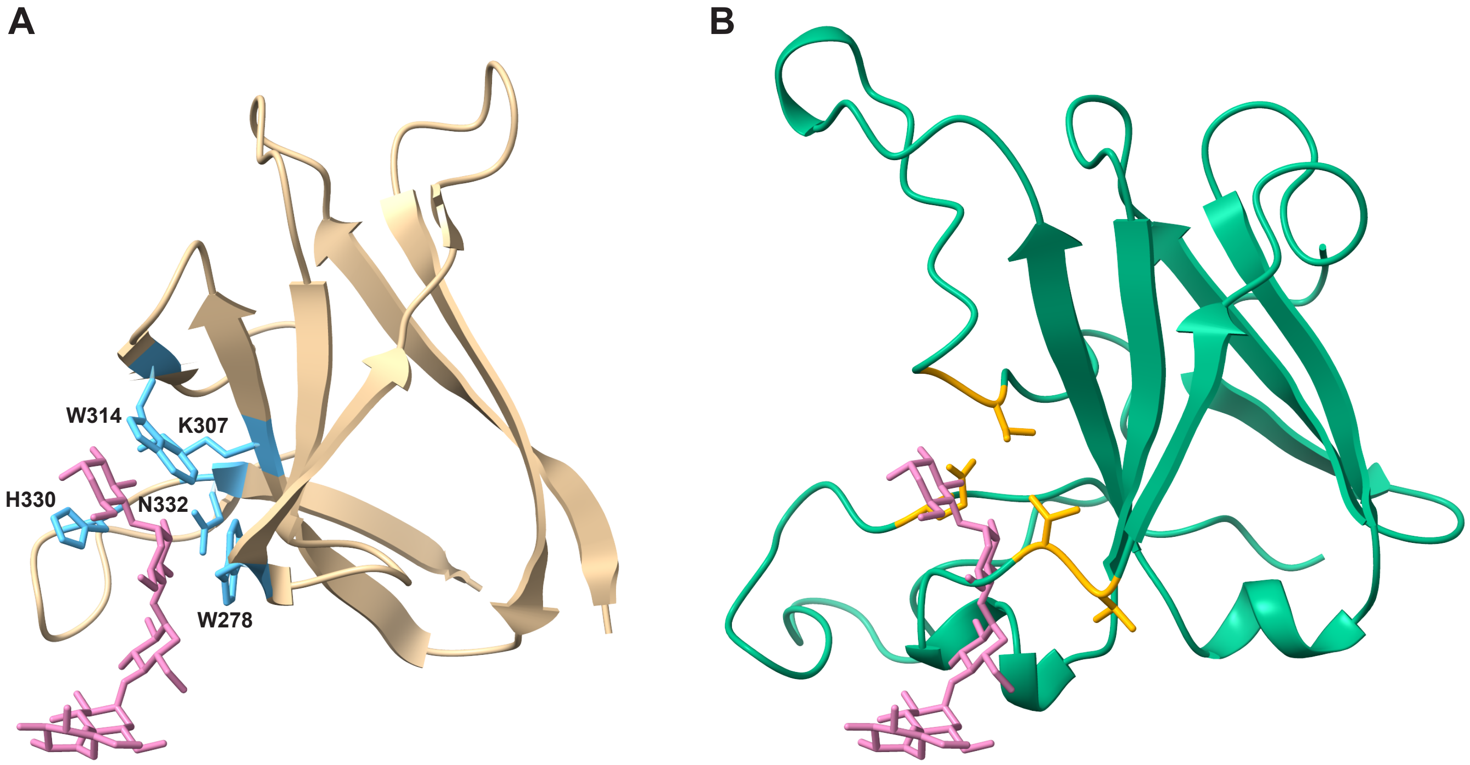

Supplementary Figure S11. Comparison of CBM48 structure between maize ISA1 and Arabidopsis SEX4 (Supports Figure 12). **A)** Crystal structure of the glucan binding site in SEX4 (Protein Data Bank identifier 4PYH). Sky blue color indicates glucan-binding residues. Reddish purple color indicates bound glucan. **B)** The CBM48 domain from the maize ISA1 AlphaFold3 structure was aligned with the crystal structure of that domain from SEX4. Glucan bound within SEX4 is overlaid into the ISA1 model. Orange color indicates the ISA1 residues located at the positions of the glucan binding residues in SEX4. An α-helix occupies the space where glucan binds to SEX4.

Supplementary Tables

Supplementary Table S1. Seed stocks available for distribution. Ear derivation is shown in Supplementary Figure S3.

Inbred	Seed Stock	Genotype
W64A	2021 pool	Non-mutant standard
W64A	21-8180-4	*isa2-339/isa2-339*
W64A	21-8149-2	*su1-Ref/su1-Ref*
W64A	21-8192-2	*isa2-339/isa2-339, su1-Ref/su1-Ref*
W64A	21-8154-5	*su1-am/su1-am*
W64A	16-4165-4	*isa2-339/isa2-339, su1-am/su1-am*
W64A	21-8161-2	*su1-st/su1-st*
W64A	21-8166-3	*isa2-339/isa2-339, su1-st/su1-st*
W64A	21-8173-6	*su1-Bn2/su1-Bn2*
W64A	21-8175-5	*isa2-339/isa2-339, su1-Bn2/su1-Bn2*
Ia453Su1+	20-8129-6	Non-mutant standard
Ia453Su1+	20-8180-6	*isa2-339/isa2-339*

Supplementary Table S2. PCR primers for amplification and sequencing of the *su1* genomic locus. Coordinates refer to inbred B73 gene model Zm00001eb174590 from maize genome assembly Zm-B73-REFERENCE-NAM-5.0.

Code	Sequence (5' → 3')	Strand	Start Position	End Position	Fragment Length
1F	CACTCCACTCGAACGCACTA	Forward	150	169	847
1R	GTTGGACACATCGTAGTA	Reverse	996	979	
2F	ATCCCCTGCTCAACCGAA	Forward	863	880	808
2R	CCTGGGTGTTTTGTCTTGCT	Reverse	1670	1651	
3F	ATTGGTTCTGTTACTTAG	Forward	1441	1458	841
3R	CTGATGGCATCTATATAC	Reverse	2281	2264	
4F	TACTTCAGCTCCTCTTCG	Forward	2224	2241	846
4R	AGCAATTTAACAGAAGTT	Reverse	3069	3052	
5F	GGGAGAGTTTTATAATTA	Forward	2949	2966	835
5R	TCCTAATGCTTAGGGGCA	Reverse	3783	3766	
6F	ACCACTTATTGACATGAT	Forward	3529	3546	836
6R	AGATCTCAGGGAATTTCT	Reverse	4364	4347	
7F	AGCCTTTTCCATGGCTCA	Forward	4283	4300	851
7R	TAGCATGTGTACACGTAT	Reverse	5133	5116	
8F	TTGGTTGATTATGATTCT	Forward	5038	5055	839
8R	AGTTATTTGTGTTTTCTG	Reverse	5876	5859	
9F	TTTTATGTGGGTTGAGCA	Forward	5823	5840	878
9R	CTTCCTCCCCCCTGCCTG	Reverse	6700	6683	
10F	CAATTAGGTGGATTAGTG	Forward	6615	6632	440
10R	ATGAAACTCTAAAGTGCG	Reverse	7054	7037	
11F	AGGCTTTTTATGTTAGTC	Forward	6877	6894	848
11R	CTAGAAGATTACAAGAAT	Reverse	7724	7707	
12F	AATATGGTCACACAAAGG	Forward	7601	7618	938
12R	CCCAACATTTTCCAGTTT	Reverse	8538	8521	

Supplementary Table S3. PCR primers for mutagenesis of the ISA1 coding sequence

Code	Sequence (5' → 3')	Amino acid substitution
su1-am mutF	TTTTCAGCCCGATGGCAataTATAGTTCCTC	R308I
su1-am mutR	GAGGAACTATAtatTGCCATCGGGCTGAAAA	
su1-NC mutF	TCGCGACACCGTGtGTCAGTTTATCAA	R504C
su1-NC mutR	TTGATAAACTGACaCACGGTGTCGCGA	
su1-SW mutF	TATAATTCGAAATACAgcCTGAGCAACG	N561S
su1-SW mutR	CGTTGCTCAGgcTGTATTTCGAATTATA	
su1-Ref mutF	ATCACAACCTGTCTcGGAATTGCGGCGA	W578R
su1-Ref mutR	TCGCCGCAATTCCgAGACAGGTTGTGAT	
su1-Bn2 mutF	AAAGGCGGTAAgAATAACACGTACTGT	N628K
su1-Bn2 mutR	ACAGTACGTGTTATTcTTACCGCCTTT	
